# Photoactivated adenylyl cyclase in cortical astrocytes promotes synaptic potentiation and reveals alterations in Huntington’s disease

**DOI:** 10.1016/j.isci.2025.113640

**Published:** 2025-09-24

**Authors:** Laia Sitjà-Roqueta, Neville M. Ngum, Evgenii A. Zherebtsov, Melike Küçükerden, Maryam Givehchi, Valentina Bova, Francis Delicata, Elena Anaya-Cubero, Enrique Santamaria, Joaquín Fernández-Irigoyen, Sara Conde-Berriozabal, Anna Castañé, Sergei Sokolovski, Edik Rafailov, Manuel J. Rodríguez, Jordi Alberch, Deniz Dalkara, Andreas Möglich, Alexander Bykov, Igor Meglinski, H. Rheinallt Parri, Mercè Masana

**Affiliations:** 1 Department of Biomedical Sciences, Institute of Neurosciences, School of Medicine and Health Sciences, Universitat de Barcelona, Barcelona, Spain; 2 Institut d’Investigacions Biomèdiques August Pi i Sunyer (IDIBAPS), Barcelona, Spain; 3 Centro de Investigación Biomédica en Red sobre Enfermedades Neurodegenerativas (CIBERNED), Madrid, Spain; 4 Aston Institute of Membrane Excellence, and College of Health and Life Sciences, Aston University, Birmingham B4 7ET, UK; 5 University of Oulu, Optoelectronics and Measurement Techniques Unit, Oulu, Finland; 6 Proteomics Platform, Clinical Neuroproteomics Unit, Navarrabiomed, Hospitalario Universitario de Navarra (HUN), Navarra Institute for Health Research (IdiSNA), Universidad Pública de Navarra (UPNA), Irunlarrea 3, 31008 Pamplona, Spain; 7 Universitat de Vic-Universitat Central de Catalunya (UVIC-UCC), IRIS-CC, Vic, Spain; 8 Aston University, College of Engineering & Physical Sciences, Birmingham, UK; 9 Sorbonne Université, INSERM, CNRS, Institut de la Vision, 17 rue Moreau, 75012 Paris, France; 10 Universität Bayreuth, Bayreuth, Germany

**Keywords:** Neuroscience, Model organism

## Abstract

Coordinated neuron-astrocyte interactions are crucial for synaptic plasticity and brain function. Cyclic adenosine monophosphate (cAMP) pathways have a key role in modulating plasticity and are disrupted in neurodegenerative diseases. Yet, the role of astrocytic cAMP remains unclear. We addressed this by expressing the photoactivatable adenylyl cyclase DdPAC in cortical astrocytes, enabling cAMP synthesis under red light stimulation. Using electrophysiological and comprehensive proteomic analyses, we determined its effects in wild-type mice. The modulation of astrocytic cAMP triggered long-term synaptic potentiation and rapidly induced the phosphorylation of proteins involved in synaptic transmission, including PKA. In Huntington’s Disease (HD) models, DdPAC activation in cortical astrocytes differentially enhanced brain hemodynamics and induced motor learning, while specifically increasing grooming and impairing coordination in HD mice. Thus, we reveal a mechanism of astrocyte-driven plasticity mediated by cAMP elevation and underscore the alterations in astrocytic cAMP signaling associated with HD.

## Highlights

• Photoactivation of DdPAC in cortical astrocytes induces synaptic potentiation
• The effects of DdPAC activation are PKA-dependent and involve glutamate signaling
• DdPAC activation in cortical astrocytes enhances cerebral blood flow
• Repeated DdPAC activation differentially modulates motor phenotype in WT and HD mice

## Graphical abstract

**Figure:**
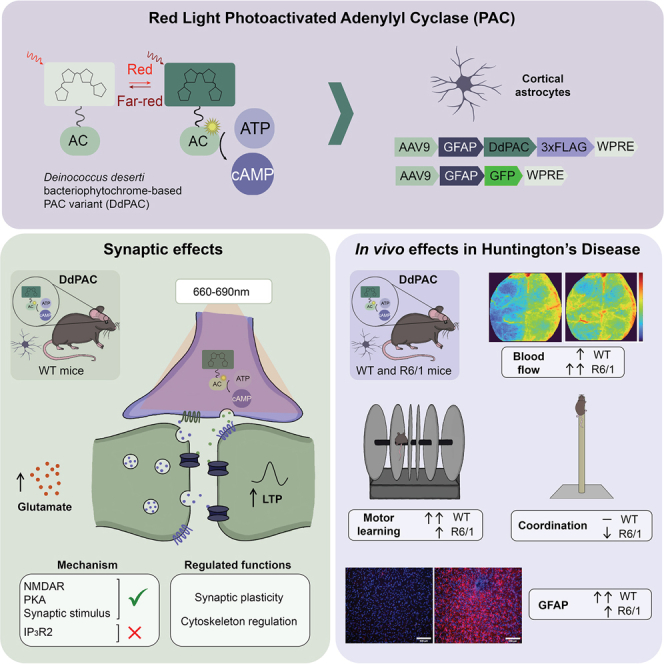

## Introduction

Astrocytes are involved in a range of processes in the brain, including energy metabolite supply, ion homeostasis, neurotransmitter clearance, release of gliotransmitters, neurovascular coupling, immune response, and behavior modulation.^1^^,^^2^^,^^3^^,^^4^ Notably, concerted neuron-astrocyte activity plays a key role in shaping neuronal plasticity.^5^^,^^6^ Recent studies also suggest that astrocytes acting at the circuit level significantly influence motor behavior.^7^^,^^8^ However, the signaling pathways underlying their diverse functions remain largely unknown.

Along with calcium, cyclic adenosine monophosphate (cAMP) is a major second messenger crucial for intracellular signaling in brain cells and makes a significant contribution to synaptic plasticity.^9^^,^^10^^,^^11^ Although most studies investigating cAMP and plasticity focus on neurons, recent research has highlighted the involvement of cAMP in hippocampal astrocytes with regard to cognition and memory.^4^ Astrocytes express neurotransmitter receptors coupled to Gs- and Gi-coupled G-protein coupled receptors (GPCRs), respectively increasing or decreasing cAMP levels. The direct activation of these astrocyte pathways using DREADDs (Designer receptors exclusively activated by designer drugs) has revealed that Gi-DREADD activation in the hippocampus affects plasticity and learning,^12^ whereas Gs-DREADD activation does not impact prefrontal cortex-dependent contextual memory.^13^^,^^14^

Altered astrocytic cAMP signaling has been observed in neurodegenerative disorders such as Huntington’s Disease (HD).^15^^,^^16^ HD is a genetically inherited neurodegenerative disorder with severe motor symptoms^17^ and prominent cortico-striatal circuitry alterations.^18^ In HD, astrocytic morphology and function around synapses are disrupted.^19^^,^^20^ Interestingly, the modulation of striatal astrocyte GPCR signaling shows differential responses between wild-type (WT) and HD mice at the levels of genes, signaling pathways, and behavior.^20^ Thus, further insight into how cAMP signaling in astrocytes differentially modulates WT and HD responses could uncover novel pathological mechanisms.

To investigate how cAMP signaling in astrocytes influences molecular and synaptic signaling and behavior, we utilized engineered photoactivated adenylyl cyclases (PACs).^21^^,^^22^ PACs are photoreceptors that enable temporal control of cAMP increase using light, in a time-dependent manner, and provide a step forward in the optogenetic control of cellular activity. *In vivo* applications of blue-light-activated PACS have demonstrated that cAMP elevation in hippocampal astrocytes induces synaptic plasticity and modulates cognition in mice, although the underlying mechanisms remain unclear.^4^ Further PAC engineering led to the development of red-light-sensitive PACs, such as the *Deinococcus deserti* bacteriophytochrome-based variant, DdPAC.^22^ This variant’s ability to be deactivated by far-red light, along with the superior tissue penetration of red wavelengths, makes DdPAC highly suitable for *in vivo* applications.

Here, we evaluated the role of astrocytic cAMP in synaptic plasticity mechanisms by selectively expressing DdPAC in cortical astrocytes and assessing red-light-induced responses through multielectrode array recordings, proteomics, and phospho-proteomics in wild-type mice. Then, we explored the potential of cortical DdPAC modulation in astrocytes to induce hemodynamic responses and affect motor behavior in both wild-type and the R6/1 mouse model of HD-. Altogether, our findings demonstrate that astrocytic cAMP signaling enhances neuronal plasticity via the phosphorylation of synaptic transmission-associated proteins and induces distinct brain and behavioral responses in WT and HD mice.

## Results

### Selective light-mediated induction of cyclic adenosine monophosphate signaling in astrocytes using DdPAC induces glutamate release and synaptic potentiation

**Figure 1:**
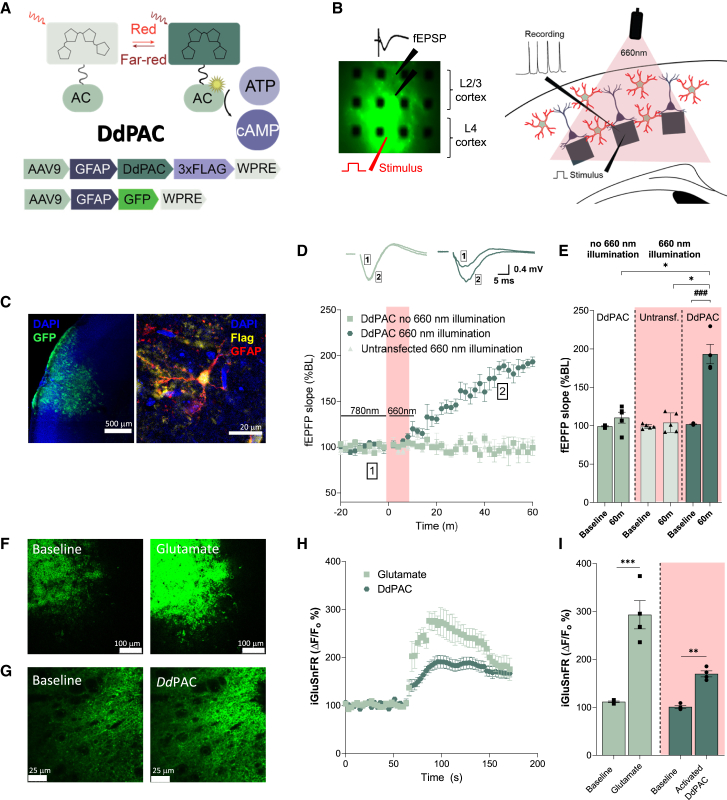
Selective light activation of DdPAC in cortical astrocytes induces synaptic plasticity (A) Schematic representation of DdPAC and its selective activation by red and inactivation by far-red light. A conformational change in the photoreceptor domain induces the activation of the adenylate cyclase in the catalytic domain, leading to cAMP increase. Bottom panel shows AAV9 constructs for DdPAC and GFP expression as a control. (B) Experimental setup: DdPAC and GFP AAV constructs were co-injected in layer 2/3 barrel cortex of wild-type mice to facilitate visualisation and correct placement on the MEA probe for recording. Left: Example of fluorescent slice image on the MEA. Right: Recording setup showing stimulation of layer 4 and evoked field potential recording in the layer 2/3 from acute slices with 660 nm light illumination. (C) Immunostaining of GFAP, DdPAC (flag), GFP, and DAPI in a mouse slice, revealing the co-localization DdPAC (flag) in astrocytes (GFAP) (4 slices from different mice analyzed). Scale bar = 500 μm (left image), 20 μm (right image). (D) Relative fEPSP slope (normalized to baseline values) versus time in 660 nm light activated DdPAC astrocytes (*n* = 5), in 660 nm light stimulation in untransfected slices (*n* = 5) and in non-illuminated slices expressing DdPAC in astrocytes (*n* = 4). (Inset) Representative traces show fEPSP at baseline (1) and 60 min (2) following 660 nm DdPAC illumination (right) or 660 nm illumination in untransfected slices (left). Symbols indicate mean ± SEM of values from the different slices at each time point. (E) Average fEPSP slope showing baseline and DdPAC activated recordings. Each point represents data from a separate slice at 60 min post stimulation (n = 4–5). For comparison between DdPAC stimulation and baseline in MEA recordings, a paired Student’s t test was used (^#^^##^*p* < 0.001). Differences between the two independent groups were determined by the Mann-Whitney test (∗*p* < 0.05). (F) Representative images show the expression of iGluSnFR in neuronal cell membranes and an increase in iGluSnFR signals following the application of 100 μM glutamate. Scale bar = 100 μm. (G) Representative images show the expression of iGluSnFR in neuronal cell membranes and an increase in iGluSnFR signals following the stimulation of DdPAC expressed in astrocytes with 660 nm light. Scale bar = 25 μm. (H) Plot of iGluSnFR ΔF/F0 at different time points where each point is the mean ± SEM for data from *n* = 4 different slices. Square symbols show response to 100 μM glutamate. Hexagon symbols show response to DdPAC activation. (I) Bar chart illustrating the change in iGluSnFR [ΔF/F0] values after the application of 100 μM Glutamate or 660 nm stimulation of DdPAC. Each point represents data from a separate slice (∗∗*p* < 0.01 and ∗∗∗*p* < 0.001, paired Student’s t test).

Photoactivated adenylyl cyclases (PAC) are potent optogenetic tools that elevate cellular cAMP levels upon stimulation by light. We used the recently developed DdPAC, a bacteriophytochrome photoreceptor that catalyzes the synthesis of cAMP when activated by red light. Additionally, it is inactivated by far-red light and so has the attributes to be used as an optogenetic tool^21^^,^^22^ (Figure 1A). DdPAC was encapsulated in an AAV9 construct under a GFAP promoter to achieve selective expression in astrocytes. A 3xFlag Tag was also included (AAV9-GFAP-DdPAC-3xFlag-WPRE, AAV-GFAP-DdPAC) to enable post-experiment cellular expression localization (Figure 1A).

As a first step to establish whether the light activation of DdPAC in astrocytes could affect neuronal plasticity, recordings of field excitatory postsynaptic potentials (fEPSPs) were conducted in acute cortical slices (Figure 1B). The DdPAC construct was co-injected with an AAV9-GFAP-eGFP-WPRE construct (AAV-GFAP-GFP), and the expression of eGFP was used as a guide to locate potential areas of DdPAC co-expression. The selective expression of DdPAC in cortical astrocytes was confirmed in a subset of slices by immunohistochemical staining for GFAP and 3xFlag (Figure 1C).

The electrical stimulation of cortical L4 resulted in a fEPSP response in cortical L2/3 that, when stimulated every 120s, displayed a consistent amplitude and slope for over 80 min. In recordings from areas expressing DdPAC, illumination with 660 nm resulted in an increase in fEPSP slope indicative of synaptic potentiation (Figures 1D and 1E). The potentiation was sustained for at least 60 min, so we have termed it DdPAC long term potentiation (DdPAC-LTP). Both 660 nm illumination of control un-transfected slices and no illumination in slices with DdPAC expressing astrocytes did not result in potentiation (Figures 1D and 1E).

To further understand how the light activation of DdPAC in astrocytes could mediate the observed synaptic potentiation,^23^ we asked whether astrocyte DdPAC activation resulted in glutamate release. The genetically encoded glutamate sensor iGluSnFr under a neuronal promoter (AAV-hSyn.iGluSnFr) was co-injected with AAV-GFAP-DdPAC. After 3 weeks, acute slices were prepared and iGluSnFR fluorescence was imaged using confocal microscopy (460 nm excitation, 530–565 nm emission). Bath application of glutamate (100 μM) resulted in a 309.1 ± 32.7% (*n* = 4) increase in fluorescence confirming the glutamate sensitivity of expressed iGluSnFR (Figures 1F–1I). DdPAC activation with a 660 nm LED also resulted in iGluSnFR fluorescence increase of 167.70 ± 4.99% (*n* = 4). These results establish that astrocyte DdPAC activation has a signaling effect following light activation and leads to glutamate release which is able to activate receptors on neighboring neurons.

### Astrocyte DdPAC-induced LTP shares properties of theta burst-LTP, is PKA dependent, IP_3_R2 independent, and requires synaptic activity

**Figure 2:**
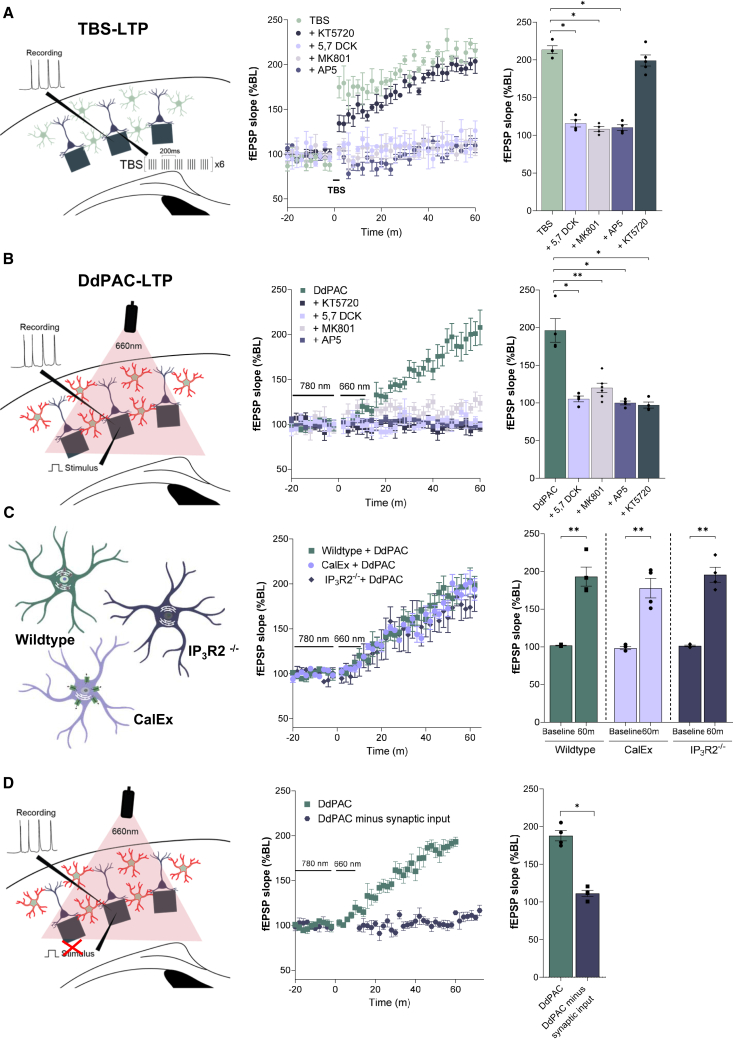
Light activated DdPAC-induced synaptic potentiation (DdPAC-LTP) shares properties of theta burst-induced potentiation (TBS-LTP) (A) Theta-burst stimulation induced LTP in cortical slices. Left panel shows a schematic representation of the experimental setup detailing TBS in layer 4 barrel cortex and recording of evoked field potentials in layer 3/4. Middle panel shows the induction of TBS-LTP in the presence of PKA inhibitor (KT5720), NMDA receptor blockers (5,7 DCK, MK801, and AP5). Right panel summarizes the fEPSP slope effects at 60 min in the different conditions. (B) DdPAC stimulation by 660 nm light increases the slope of evoked fEPSPs. Left panel shows a schematic representation of the experimental setup detailing 660 nm illumination, input stimulation (1 Hz) in layer 4 barrel cortex, and recording of evoked fEPSPs in layer 3/4. Middle panel shows the induction of fEPSP potentiation by 660 nm light and in the presence of PKA inhibitor (KT5720), NMDA receptor blockers (5,7 DCK, MK801, and AP5). Right panel shows a summary of the effects on the fEPSP slope. (C) 660 nm light evoked fEPSP in cortical slices expressing DdPAC in WT, IP_3_R2^−/−^ and CalEX mice. Left panel shows a schematic representation of the astrocytic characteristics of the mouse lines used for MEA recording. Middle panel shows the induction of fEPSP slope potentiation by 660 nm light in the different mouse lines. Right panel shows a summary of the fEPSP slope potentiation effects. (D) 660 nm light evoked field potentials in cortical slices expressing DdPAC in WT in the presence or absence of 1 Hz input during MEA recordings. Middle panel shows the induction of fEPSP slope potentiation by light. Right panel shows a summary of the fEPSP slope potentiation effects. For A–D, Middle panels show plots of fEPSP slope at experimental time points where symbols are mean ± SEM for experiments in separate slices (n = 4–5), with symbols corresponding to indicated experimental conditions. Panels on the right show summary bar graphs of mean ± SEM (n = 4–5) of normalised fEPSP slope 60 min post stimulation, where each point represents data from a single slice. For comparison between LTP and baseline in MEA recordings, paired Student’s t test was used (∗∗*p* < 0.01). Differences between the two independent groups were determined by the Mann-Whitney test (∗*p* < 0.05 and ∗∗*p* < 0.01).

To determine the mechanism of astrocyte DdPAC-LTP, we compared it to conventional electrical synaptic stimulation-induced LTP (Figure 2A). Theta burst stimulation (TBS) was used as an established protocol for inducing LTP in cortical L4-L2/3 synapses.^24^ TBS-induced LTP (TBS-LTP) lasted for over 60 min (Figure 2A), with a magnitude similar to DdPAC-LTP (Figure 2B) (TBS: 214.80 ± 5.56%, *n* = 4, DdPAC: 187.90 ± 6.92%, *n* = 4, *p* = 0.057), although both forms had their own distinctive kinetic profiles (Figures 2A and 2B). TBS-LTP and DdPAC-LTP induction were blocked by the presence of AP5, indicating a requirement for NMDA receptor (NMDAR) activation (Figures 2A and 2B). Furthermore, DdPAC-LTP and TBS-LTP induction were also blocked in the presence of MK801, an open channel blocker, and 5,7 DCK, an antagonist of the NMDAR Glycine/Serine site (DdPAC MK801: 104.90 ± 1.36, *p* = 0.049, TBS MK801: 119.95 ± 6.32, *p* = 0.029, and DdPAC 5,7 DCK: 102.30 ± 3.15, *p* = 0.029, TBS 5,7 DCK: 108.32 ± 3.84, *p* = 0.028) (Figures 2A and 2B). These results indicate that both DdPAC-LTP and TBS-LTP require NMDAR ionotropic signaling and co-agonist activation of the Glycine/D-Serine site. Since DdPAC activation catalyses the formation of cAMP, we then tested the role of protein kinase A (PKA) as one of the main targets of cAMP. DdPAC-LTP induction was abrogated in the presence of PKA inhibitor KT5270 (96.67 ± 4.09%, *n* = 4, *p* = 0.002), but TBS-LTP induction was unaffected (198.90 ± 7.24%, *n* = 5, *p* = 0.52) (Figures 2A and 2B).

The recognition of the role of astrocytes in LTP in the cortex, hippocampus, and other brain areas is increasing.^24^^,^^25^^,^^26^ This role often depends on increases in intracellular Ca^2+^ levels within astrocytes.^26^^,^^27^^,^^28^ We therefore sought to address this in the case of DdPAC-LTP. Therefore, DdPAC was expressed in the cortex of the IP_3_R2^−/−^ mice (Figure 2C). The R2 subtype of the IP_3_ receptor is the most expressed in astrocytes and an important mediator of Ca^2+^ signaling.^29^^,^^30^ In the mutant IP_3_R2^−/−^ mouse, spontaneous and evoked astrocyte calcium signaling is greatly reduced.^24^^,^^31^ 660 nm stimulation resulted in LTP in both IP_3_R2^+/+^ and IP_3_R2^−/−^ (176.33 ± 12.29%, *n* = 4, *p* > 0.99), indicating a lack of a role for IP_3_R2 signaling. To further address the role of calcium, we expressed the plasma membrane Ca^2+^ extrusion pump isoform PMCA2w/b in astrocytes, developed by Khakh and colleagues^32^ and named CalEx (Figures 2C and S1). The co-expression of CalEx and DdPAC still resulted in DdPAC-LTP in response to 660 nm stimulation (195.98 ± 9.50%, *n* = 4, *p* > 0.99) (Figure 2C). These results suggest a lack of dependence of the DdPAC effect on astrocyte Ca^2+^ signaling, but do not rule out completely a Ca^2+^ contribution not affected by CalEx.

The finding that the DdPAC-LTP induction mechanism shares pathways with electrically induced activity-dependent TBS led us to further characterise the properties of DdPAC-LTP (Figure S2). When TBS was applied in DdPAC expressing slices in conjunction with 660 nm light stimulation, there was no increase in the degree of LTP (TBS-LTP: 214.80 ± 5.56%, *n* = 3, DdPAC-LTP: 187.90 ± 6.92%, *n* = 4, and 201.5 ± 9.36% for TBS + DdPAC-LTP *n* = 5, *p* = 0.58) (Figure S2A). TBS stimulation applied following the 660 nm induction of DdPAC-LTP also did not further increase potentiation (Figure S2B). Therefore, TBS-LTP and DdPAC-LTP are not additive, which is consistent with the respective LTP induction being through common mechanisms.

LTP and LTD are considered opposing mechanisms in the brain that act to modify and balance synaptic strength.^33^ In L4-L3 synapses low frequency stimulation (LFS) of 900 stimuli at 1Hz induces synaptic depression (Figures S2C and S2D). If DdPAC-LTP shares a mechanism with electrically induced TBS-LTP, then electrically induced LFS should be able to reverse it. Indeed, this was the case (Figure S2C). This means that electrical synaptic activity can reverse the effect of DdPAC cAMP-mediated light activation.

It is known that TBS-LTP and LFS-induced LTD (LFS-LTD) utilize some of the same cellular mechanisms, including NMDAR and astrocyte Ca^2+^. We therefore tested whether 660 nm DdPAC activation would enhance or abrogate LFS-induced LTD. In fact, when they were co-applied, 660 nm DdPAC activation counteracted the effect of LFS and instead induced DdPAC-LTP (LFS-LTD: 55.65 ± 2.53%, *n* = 4, versus induced DdPAC-LTP: 184.02 ± 19.26%, *n* = 4, *p* = 0.029) (Figure S2D).

We then examined whether 660 nm stimulation alone could induce a synaptic change that resulted in LTP. Following a period of baseline synaptic stimulation at 0.008Hz, this stimulation was paused, and DdPAC was activated by 660 nm light for 10 min. After 660 nm light stimulation cessation, baseline stimulation was resumed. Against expectations, there was no potentiation of the subsequent synaptic responses (111.23 ± 4.12%, *n* = 4, *p* = 0.014) (Figure 2D). The results indicate two things: that astrocyte PKA-dependent LTP induction requires the co-activation of astrocyte pathways and likely postsynaptic depolarization, and that the effect of DdPAC and PKA does not last beyond the 660 nm light stimulus. DdPAC light activation enhanced synaptic activity at 1 Hz and 0.008 Hz, but not at 0 Hz. This indicates that while DdPAC-induced LTP requires synaptic activity, unlike TBS-LTP, it is synaptic frequency-independent. This further indicates that DdPAC activation is a potentially powerful tool to induce potentiation in active networks by exposure to red light.

### *In vivo* astrocyte DdPAC stimulation displays a broad impact on synaptic transmission phosphoproteins within minutes

**Figure 3:**
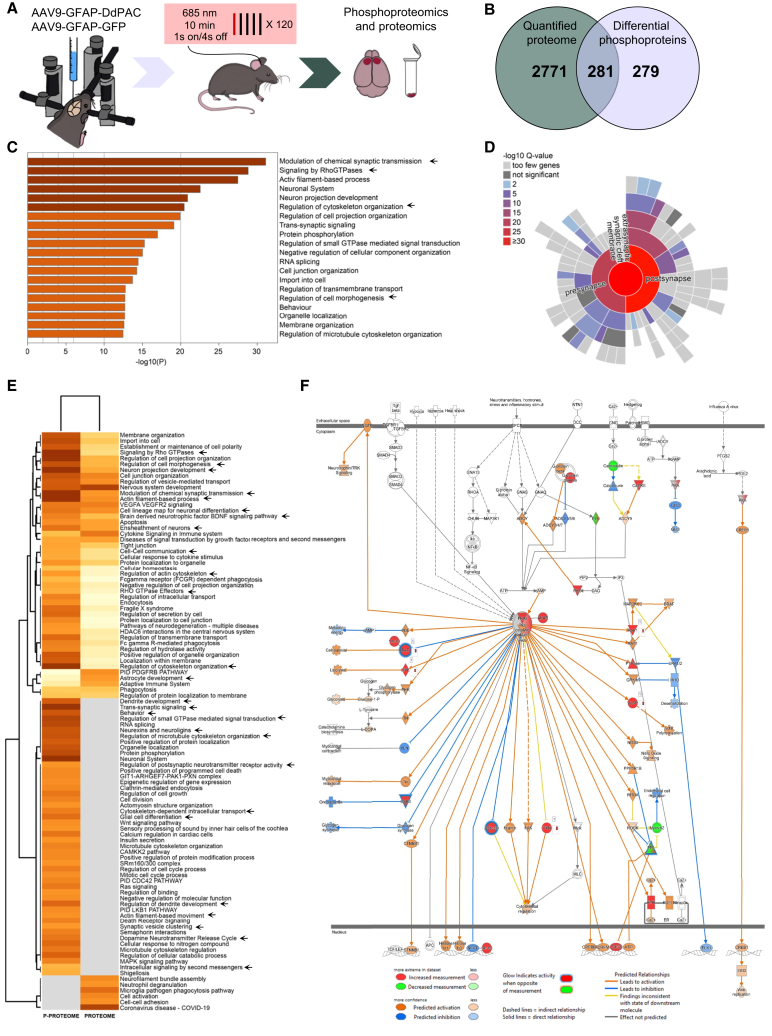
Astrocytic DdPAC stimulation phospho-proteome confirms the activation of PKA-dependent pathways and reveals synaptic plasticity modulation (A) Schematic representation of experimental design. Injection of AAV-GFAP-DdPAC or AAV-GFAP-GFP as control was performed in the M2 cortex of WT mice (*n* = 4 each). DdPAC stimulation was performed in freely moving mice using a 685 nm light source for 10 min (1 s light ON and 4 s light OFF cycle). Frontal cortex brain tissue was dissected immediately after *in vivo* light delivery. (B) Number of quantified proteins and differential phosphoproteins. (C) Top 20 main phospho-proteome ontologies. (D) Main synaptic sub-cellular localization of the phospho-proteome. (E) Heatmap showing the main pathways revealed by the phospho-proteome (left) and proteome (right). (F) PKA network analysis obtained from the phospho-proteome dataset.

To elucidate the molecular targets underlying the DdPAC potentiating response, we conducted phosphoproteomics and proteomics analyses (Figure 3 and S3). Frontal cortex tissue was obtained immediately after the *in vivo* light stimulation of DdPAC or GFP expressed in cortical astrocytes (Figure 3A). The changes induced by DdPAC stimulation were stronger in the phospho-proteome than the proteome dataset (Figures 3B, S3A, and S3B). In detail, ontology analyses of the phospho-proteome confirmed that DdPAC stimulation in astrocytes leads to the phosphorylation of synaptic plasticity associated proteins (Figure 3C). Interestingly, the top-modulated ontologies include the modulation of chemical synapses, followed by signaling by Rho GTPases, which has been reported to modulate actin dynamics,^34^ a finding that also consistently appears in the analysis. Moreover, the most abundant phosphoprotein changes were localized at the pre- and post-synapse, including the synaptic cleft (Figure 3D), accordingly with its involvement in synaptic plasticity mechanisms. In turn, the proteome dataset (Figures S3B–S3D) includes ontologies related to synaptic transmission, neuron projection development, BDNF signaling, *trans*-synaptic signaling, postsynaptic neurotransmitter receptor activity, among others. All these changes are indicative of abundant synaptic changes within minutes, as expected from the observed DdPAC-induced synaptic plasticity from slice experiments.

Moreover, PKA network analyses (Figure 3D) confirmed that *in vivo* DdPAC activation in astrocytes operates through PKA signaling. First, cAMP-dependent protein kinase type I-alpha regulatory subunit (PRKAR1A) and A-kinase anchor protein 8 (AKAP8) were found to be more phosphorylated in DdPAC-stimulated cortical samples compared to control. Additionally, many PKA downstream effector proteins were also phosphorylated, such as the actin-binding protein Adducin, the focal adhesion kinase-associated protein Paxillin (PXN), and Vasodilator-stimulated phosphoprotein (VASP), all involved in actin and cytoskeletal modulation.^35^^,^^36^^,^^37^

Finally, while phospho-proteomic and proteomic differential proteins include proteins associated with astrocyte development, which are indicative of cell-autonomous effects, the impact of DdPAC stimulation was observed beyond the astrocyte cell type. Analysis of cell-type markers^38^ shows phosphoprotein changes in proteins enriched not only in astrocytes, but also in neurons, microglia, and oligodendrocytes. This indicates that DdPAC activation in astrocytes has a broad impact involving a wide variety of responses in diverse brain cells in just a few minutes.

### Astrocyte DdPAC stimulation generates distinct and widespread hemodynamic increases in the cortex of wild type and Huntington’s disease mice

**Figure 4:**
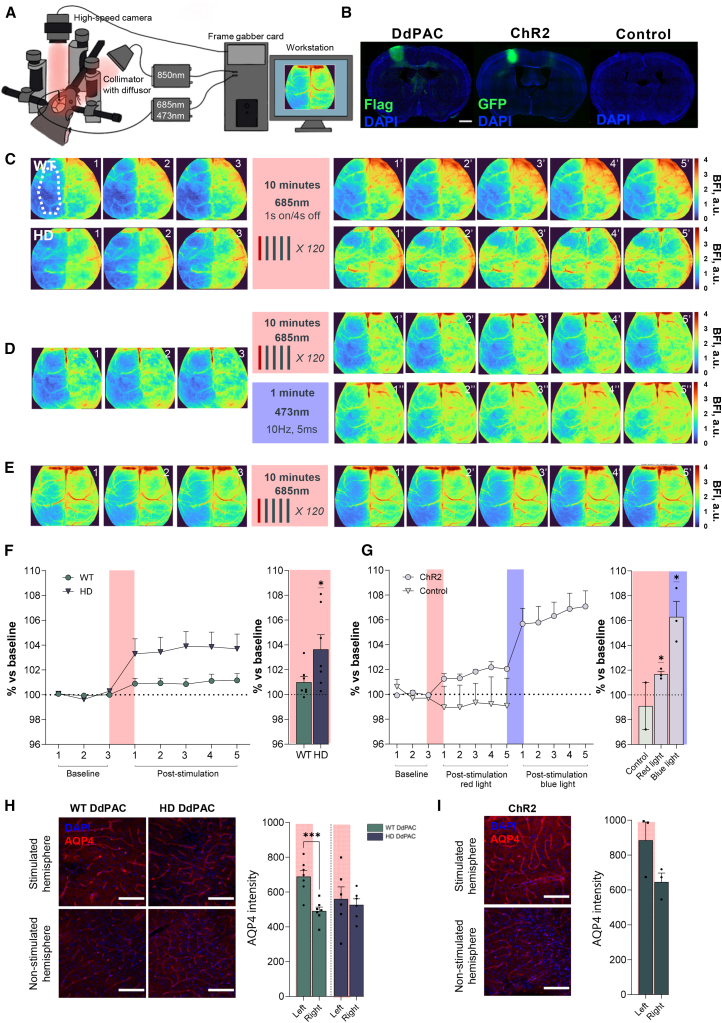
Astrocytic DdPAC stimulation induces distinct cortical hemodynamic changes in WT and HD mice (A) Imaging setup for mapping light dependent cortical blood flow changes obtained with DWS in a head-fixed anesthetized mouse. (B) Validation of the expression of DdPAC and opsins (ChR2) in astrocytes and neurons, respectively, in the cortex from the left hemisphere was performed in all mice. Naive mice were used as a control. Representative immunofluorescence shows virus constructs (green) and DAPI (blue) staining (Scale bar 1 mm). (C) Color-coded brain images indicating cortical blood flow before (1, 2, 3) and after red light (1′, 2′, 3′, 4′, 5′) stimulation in DdPAC-expressing WT (top images) and the R6/1 mouse model of HD (bottom images). (D) Color-coded brain images indicate cortical blood flow before (1, 2, 3) and after (1′, 2′, 3′, 4′, 5′) red (top) and blue (1″, 2″, 3″, 4″, 5″, bottom) light stimulation in WT mice expressing opsins (ChR2) in neurons. (E) Color-coded brain images indicate cortical blood flow before (1, 2, 3) and after (1′, 2′, 3′, 4′, 5′) red light stimulation on control (naive) WT mice. (F) Quantification of the percentage of hemodynamic change induced by DdPAC in the left hemispheres in WT and the R6/1 mouse model of HD for each image (left) and mean post stimulation percentage of change versus baseline (right). On the left panel, circles represent mean ± SEM values from WT DdPAC (*n* = 7) and triangles represent mean ± SEM values from HD DdPAC (*n* = 7). Individual dots on the right panel show the mean post stimulation value for each mouse. (G) Quantification of the percentage of hemodynamic change induced by red and blue light in mice expressing ChR2 and control (left) and mean percentage change (right). On the left panel, circles represent mean ± SEM values from WT ChR2 (*n* = 3) and triangles represent mean ± SEM values from Controls (*n* = 2). Individual dots on the right panel show the mean post stimulation percentage of change versus baseline of each control mouse and during red or blue laser stimulation of each ChR2 expressing mouse. (F and G) Hemodynamic effects were analyzed by one sample T-test (∗*p* < 0.05). (H) Representative immunofluorescent images of Aquaporin4 expression (red) in light stimulated (top panels) or control hemisphere (bottom panels), from WT (left) and HD (right) expressing DdPAC (Scale bar = 100 μM). Histograms show the quantification of the AQP4 intensity in both hemispheres. (I) Representative immunofluorescent images of Aquaporin4 expression (red) in light stimulated (top panels) or control hemisphere (bottom panels) of WT expressing ChR2 (Scale bar = 100 μM). Histograms show the quantification of the AQP4 intensity in both hemispheres. Each point represents data from an individual mouse, and 2-4 sections per mouse were analyzed (WT DdPAC *n* = 7; R6/1 DdPAC *n* = 7; WT ChR2 *n* = 3; Controls *n* = 2). Differences were analyzed by paired Student T-test (∗∗∗*p* < 0.001).

Astrocytes regulate cerebral blood flow (CBF) in response to neuronal activity, although the mechanisms involved are not yet well understood. Indeed, calcium involvement in CBF responses has been controversial, and a recent study using a mouse model that can elevate calcium levels selectively in astrocytes through light-activated Gq-type GPCR concluded that Gq-GPCR-induced calcium signaling in astrocytes was not involved in the short-term control of cerebral blood flow.^39^ Because of our robust findings in synaptic protein regulation as well as on the DdPAC-LTP induction, which seemed to lack a dependence on calcium, in this study we therefore tested whether astrocyte cAMP signaling could lead to changes in cerebral blood flow. For this purpose, we developed a bespoke application of the Dynamic Light Scattering imaging technique, known as Diffusing Wave Spectroscopy (DWS),^40^ which allows the direct visualization of blood flow *trans*-cranially at the mesoscale level, enabling the acquisition of label free wide-field images from the dorsal surface of the adult mouse cortex (Figure 4). In addition, we selected an HD mouse model, where altered astrocytic function^19^^,^^41^^,^^42^ and changes in cAMP signaling^16^^,^^43^ have both been reported.

WT and HD mice expressing DdPAC in cortical astrocytes were anesthetized, head-fixed, and a small hole was made in the skull to ensure that delivered red light reached the cortex (left hemisphere) during DWS imaging (Figure 4A). After three baseline image collections, we stimulated with red light for 10 min and obtained an additional five images right after (Figure 4C). Our data showed that red light induced an increase in CBF throughout the cortex in mice expressing DdPAC in astrocytes (Figures 4C and 4F). Specifically, red light-mediated DdPAC stimulation in the left hemisphere induced a 1.5% increase in blood flow in the same hemisphere in WT mice (one sample t-test, *p* = 0.07). In contrast, in HD mice, the activation of DdPAC induced a 3.5% increase in blood flow (one sample t-test, *p* = 0.02). As a control for neuronal activity, we also expressed blue-dependent ChR2 in cortical neurons with AAV-CamKII-ChR2 in WT mice (Figures 4D and 4G). The activation of opsins with 685 nm red light induced a ∼1.6% change (one sample t-test, *p* = 0.03), while 473 nm blue light induced ∼5.8% change in blood flow (one sample t-test, *p* = 0.01). No effects were induced by red 685 nm light stimulation in control (naive) mice (one sample t-test, *p* = 0.7), indicating the specificity of the light-induced effects by opsins (Figures 4E and 4G). This set of data demonstrates that astrocytic cAMP activity induced by DdPAC can modulate cortical CBF and provides further evidence on aberrant astrocytic function in HD mice.

To further investigate the hemodynamic changes observed by DdPAC stimulation in astrocytes, we evaluated Aquaporin 4 (AQP4) expression in the cortex of stimulated animals. AQP4 is a water channel highly expressed in the endfeet of astrocytes, which facilitates water movement at the blood-brain interface.^44^^,^^45^ At the synapse, AQP4 also regulates water and potassium fluxes in astrocytes through cAMP^46^ and synaptic activity through the mobility of the astrocytic processes.^47^ Thus, we explored the levels of AQP4 expression in the DdPAC-stimulated hemisphere vs. the non-stimulated hemisphere (Figures 4H and 4I). Immunofluorescence results showed an increase in APQ4 expression in the DdPAC expressing hemisphere (left) in WT mice stimulated with red light (Paired t-test, two-tailed, *p* = 0.0002). In contrast, no differences were observed between hemispheres in DdPAC-stimulated HD mice (Paired t-test, two-tailed, *p* = 0.7). WT mice stimulated with neuronal ChR2 also did not show a change in AQP4 expression (Paired t-test, two-tailed, *p* = 0.2). These results indicate that astrocyte activity induced by DdPAC triggers local changes in AQP4 expression, which are absent in HD mice.

### Acute DdPAC activation does not alter spontaneous behavior in wild type mice but enhances stereotypies in Huntington’s disease mice

**Figure 5:**
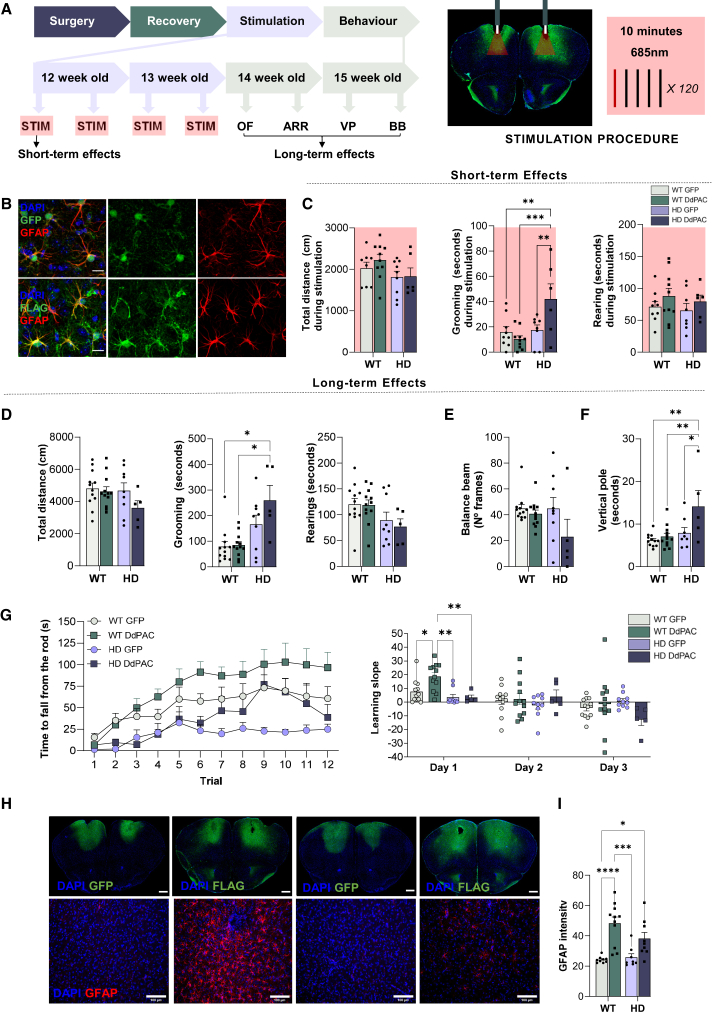
DdPAC activation in M2 cortical astrocytes differentially impacts motor behavior in WT and HD mice (A) Experimental timeline. Injection of AAV-GFAP-DdPAC or AAV-GFAP-GFP as a control was performed bilaterally in the M2 cortex of WT and R6/1 mice at 8 weeks. DdPAC stimulation started at 12 weeks and was repeated 4 times every 3 days. Behavioral tests started the day after the last stimulation with 14 weeks old mice and included open field (OF), accelerating rotarod (ARR), vertical pole (VP), and balance beam (BB). DdPAC stimulation consisted of 1 s of 685 nm light ON followed by 4 s light OFF, 5-min per hemisphere. (B) Representative immunostaining of DdPAC (flag) or GFP with GFAP and DAPI in mouse cortical slices, revealing the co-localization of DdPAC/GFP in astrocytes. 2-3 sections per animal were analyzed (scale bar 15 μm). (C) Short-term behavioral effects during the first stimulation on: locomotion, as total distance traveled; time spent grooming, as a measure of stereotypic behavior; and time spent doing rearing, as a measure of exploration time. (D) Long-term behavioral effects of DdPAC stimulation on locomotion, grooming, and rearing in the open field. (E) Effects of DdPAC stimulation on motor coordination were evaluated as the number of frames traveled in the balance beam. (F) Effects of DdPAC stimulation on motor coordination evaluated in the vertical pole, quantified with the seconds used to flip and descend the pole. (G) Effects of DdPAC stimulation on motor learning evaluated by an accelerated rotarod, as the latency to fall from the rotarod (left) and the learning slope per day and group (right). (H) Top row: representative fluorescent images showing in green the AAV-infected brain regions (GFP for AAV-GFAP-GFP; FLAG, for AAV-GFAP-DdPAC) and in blue DAPI staining (scale bar 100 μm) in WT (left) and HD mice (right). Bottom row: representative fluorescent images showing GFAP positive astrocytes in the infected region (scale bar 100 μm). Two slices per mouse and two images per slice (one for each hemisphere) were analyzed. (I) Quantification of GFAP intensity. For C–I, each point represents data from a single mouse with circles corresponding to AAV-GFAP-GFP and squares corresponding to AAV-GFAP-DdPAC expressing mouse. (C-I) Differences were analyzed by two or three-way ANOVA followed by Bonferroni post-hoc test (∗*p* < 0.05, ∗∗*p* < 0.01, ∗∗∗*p* < 0.001, and ∗∗∗∗*p* < 0.0001). WT GFP *n* = 12, WT DdPAC *n* = 13, HD GFP *n* = 10, HD DdPAC *n* = 10.

To determine whether DdPAC-stimulation could impact network functions and behavior, we conducted *in vivo* behavioral studies in WT and in the R6/1 mouse model of HD. To do so, we bilaterally injected DdPAC in cortical astrocytes of WT and the HD model and performed DdPAC stimulation four times, with two days in between each stimulation session (Figure 5A). To assess if DdPAC activation in M2 cortex was having an acute impact on behavior, we tested paradigms of locomotion, exploration and stereotypic behavior (Figures 5A–5C). During a 10 min-stimulation period, total distance walked by the animals remained unaltered (genotype: F_(1,29)_ = 3.784 *p* = 0.06; stimulation: F_(1,29)_ = 0.4822 *p* = 0.5; genotype/stimulation: F_(1,29)_ = 0.3245 *p* = 0.6). Similarly, exploratory behavior, measured by the time spent rearing, was unchanged (genotype: F_(1,29)_ = 0.4304 *p* = 0.5; stimulation: F_(1,29)_ = 0.1.941 *p* = 0.2; genotype/stimulation: F_(1,29)_ = 0.01343 *p* = 0.9). Stereotypic behavior, measured by the time spent grooming, was increased specifically in HD DdPAC mice. Two-way ANOVA revealed a significant genotype (F_(1,29)_ = 9.031 *p* = 0.005) and genotype-treatment interaction effect (F_(1,29)_ = 7.383 *p* = 0.01), although no treatment effect (F_(1,29)_ = 9.031 *p* = 0.1). Bonferroni *post hoc* showed significant differences between HD DdPAC with all other groups (vs. WT GFP *p* = 0.02; vs. WT DdPAC *p* = 0.003 and vs. HD GFP *p* = 0.04). Our data demonstrate that the modulation of cortical astrocytes has a minor impact on spontaneous behavior in WT but can generate associated behavioral responses, such as self-grooming^48^ after only a brief period of DdPAC activation in HD mice.

### Repetitive DdPAC stimulation in cortical astrocytes improves motor learning but compromises coordination, specifically in Huntington’s disease mice

We then investigated the long-term effects of repetitive DdPAC stimulation *in vivo* using well-characterized motor-related behavioral tests, which are associated with HD cortico-striatal pathology^49^^,^^50^ (Figures 5D–5G). Starting the day after the last stimulation, we again assessed locomotion, stereotypies, and exploratory behavior during an open field test (Figure 5D). Similar to the short-term effects observed by DdPAC stimulation, after four sessions of light stimulation, HD DdPAC increased grooming while locomotion and exploratory behavior remained unaltered. Two-way ANOVA showed no differences in genotype (F_(1,34)_ = 2.065 *p* = 0.2) nor stimulation (F_(1,34)_ = 2.439 *p* = 0.1), nor genotype/stimulation interaction effects (F_(1,34)_ = 1.206 *p* = 0.3) in locomotion. Exploratory behavior showed genotype (F_(1,34)_ = 6.946 *p* = 0.01) but not stimulation (F_(1,34)_ = 0.2505 *p* = 0.6) or genotype/stimulation interaction effects (F_(1,34)_ = 0.1571 *p* = 0.7). Regarding the time the animals spent grooming, we observed genotype effects (F_(1,34)_ = 20.82 *p* < 0.0001) and a tendency for the stimulation effect (F_(1,34)_ = 3.029 *p* = 0.09), but not genotype/stimulation interaction effect (F_(1,34)_ = 2.343 *p* = 0.1). Bonferroni post-hoc showed significant differences between HD DdPAC and both WT GFP (*p* = 0.002) and WT DdPAC (*p* = 0.002).

Next, we evaluated motor learning using the cortico-striatal dependent accelerating rotarod test^51^^,^^52^ (Figure 5G). We observed that HD mice display impaired motor learning compared to WT control, shown by a reduced latency to fall from the rotarod, as expected in 14-week-old mice.^53^ DdPAC stimulation improved the performance in the rotarod in both genotypes, although the effects were more prominent in WT mice. Three-way ANOVA showed trial (F_(11,385)_ = 17.47 *p* < 0.0001), genotype (F_(1,35)_ = 12.07 *p* = 0.001) and trial/stimulation interaction effect (F_(11, 385)_ = 2.662 *p* = 0.003); but not stimulation (F_(1,35)_ = 3.560 *p* = 0.07), trial/genotype (F_(11,385)_ = 1.404 *p* = 0.2), nor genotype/stimulation (F_(1,35)_ = 0.04 *p* = 0.8), trial/genotype/stimulation (F_(11,385)_ = 0.9285 *p* = 0.5). When the learning slope was investigated, we observed an improvement in learning on the first day in WT mice, as indicated by a two-way ANOVA analysis with group (F_(3,104)_ = 2.729 *p* = 0.05) and day effect (F_(1483,77.11)_ = 11.66 *p* = 0.0002), although not day/group effect (F_(6,104)_ = 2.047 *p* = 0.065). Bonferroni post hoc test revealed significant differences during the first day of stimulation between the WT DdPAC and WT GFP (*p* = 0.047), HD GFP (*p* = 0.002), and HD DdPAC groups (*p* = 0.002). This demonstrates enhanced motor learning on the first day of stimulation in WT mice.

Finally, we assessed coordination changes with the balance beam and vertical pole test, which are associated with basal ganglia function and are strongly affected at late symptomatic stages of HD mice (>16-week-old R6/1 mice). These coordination changes have been previously shown to be modulated by cortico-striatal optogenetic stimulation in HD mice^49^ (Figures 5E and 5F). In 15-week-old AAV-GFAP-GFP expressing mice, no coordination deficits were observed in either the vertical pole test or the balance beam in HD compared to WT, as expected at this stage of HD. In both tests, detrimental effects of DdPAC stimulation on coordination were observed, particularly in HD mice. The balance beam test showed stimulation effects (F_(1,34)_ = 4.237 *p* = 0.047) but no genotype (F_(1,34)_ = 1.973 *p* = 0.2), nor genotype/stimulation interaction (F_(1,34)_ = 1.960 *p* = 0.2) effects. Bonferroni *post hoc* did not show significant differences between groups. In the vertical pole test, which also evaluates coordination, stimulation (F_(1,33)_ = 7.202 *p* = 0.01), genotype (F_(1,33)_ = 11.05 *p* = 0.002) and genotype/stimulation interaction effects (F_(1,33)_ = 4.083 *p* = 0.051) were observed by a two-way ANOVA. Bonferroni *post-hoc* showed a significant increase in the total time to perform the test between HD DdPAC and the WT GFP (*p* = 0.002), HD GFP (*p* = 0.04), and WT DdPAC (*p* = 0.007) groups.

Moreover, upon the histological examination of mouse brains injected with AAVs expressing either DdPAC or reporter genes (Figure 5B), we observed changes in GFAP expression levels in the tissue of stimulated mice compared to their respective controls. Thus, we decided to analyze the intensity of GFAP by immunofluorescence (Figures 5H and 5I). We observed significantly increased GFAP expression levels in both WT and HD DdPAC-stimulated mice compared to GFP controls. This aligns with our proteomic data, which indicated that short-term DdPAC stimulation elevated GFAP levels in WT mice and indicates astrocytic activation by DdPAC. Two-way ANOVA showed the effects of the simulation (F_(1,33)_ = 29.67 *p* < 0.0001), but not genotype (F_(1,33)_ = 1.617 *p* = 0.2) or genotype/stimulation interaction (F_(1,33)_ = 3.022 *p* = 0.09). Using a Bonferroni *post-hoc* analysis, we detected a significant increase in the difference between WT GFP and WT DdPAC (*p* < 0.0001) and HD DdPAC (*p* > 0.03), and between WT DdPAC and HD GFP (*p* < 0.0002).

Overall, these findings indicate that an increase in cAMP in cortical astrocytes triggers cortico-striatal-associated behavioral responses in mice, particularly by enhancing motor learning. Notably, DdPAC stimulation produces different behavioral outcomes in WT and HD mice, improving motor learning while impairing coordination in HD mice. This suggests that alterations in astrocytic cAMP signaling in the cortex may contribute to motor symptoms in HD. Further research is needed to fully understand the role of astrocytic cAMP signaling in HD pathology.

## Discussion

In this study, we used the red-light-responsive PAC, DdPAC, to elucidate the role of astrocytic cAMP signaling in neuronal plasticity. Our findings reveal that cAMP signaling modulation in astrocytes rapidly induces a previously undescribed form of potentiation that is frequency-independent yet activity-dependent. These effects occur within minutes and are mediated through fast, complex, and converging molecular mechanisms involving glutamate release, the phosphorylation of proteins associated with synaptic function, and morphology modulation. At the network level, DdPAC-mediated cAMP modulation in cortical astrocytes increased CBF across the cortex and induced long-lasting behavioral phenotypes. Notably, our results reveal differential *in vivo* effects of DdPAC stimulation under pathological conditions, such as HD. Together, these findings underscore the broad impact of astrocytic cAMP signaling in synaptic plasticity and brain network function, while also uncovering alterations under pathological conditions.

Astrocytes play crucial roles in synaptic plasticity.^5^^,^^6^ While much research has focused on Ca^2+^ signalling-mediated events, little is understood about how astrocytic cAMP might shape plasticity and brain function. Our data showed that cAMP induction specifically in astrocytes modulates synaptic potentiation through a PKA-dependent mechanism. These results align with previous studies using non-cell specific cAMP modulators, such as forskolin, an adenylate cyclase activator, that demonstrated the involvement of cAMP in synaptic potentiation,^54^^,^^55^ perhaps also indicating an astrocyte locus in these studies. Moreover, these astrocytic cAMP signalling-dependent potentiation shares some of the mechanisms known to mediate synaptic plasticity by astrocytes, such as increases in glutamate^56^ and modulation of synaptic potentiation through NMDAR and D-serine.^25^^,^^26^ Of note, the potentiation induced by astrocytic DdPAC stimulation was only observed in the presence of synaptic activity. This is consistent with a situation where astrocyte cAMP-induced glutamate activates NMDAR and requires synaptically induced neuronal depolarization to enable NMDAR ionotropic signaling associated with classical LTP. There are multiple potential release mechanisms that could mediate the increase in glutamate following astrocyte DdPAC activation. The most likely is perhaps vesicular release,^57^^,^^58^ although associated with calcium dependency. A role for PKA might point to phosphorylation and a channel mediated process of which there are several candidates such as Volume regulated Channels SWELL1,^59^ TREK 1 channels and Best 1 channels.^60^ Alternatively, it is possible that another gliotransmitter such as ATP is released that acts on local neurons to induce neuronal glutamate release.^61^A notable finding was that, in addition to requiring activity in conjunction with DdPAC activation to induce DdPAC-LTP, the induction of LTP was frequency-independent, allowing even synaptic frequencies that normally induce LTD to instead induce LTP. It therefore appears that astrocyte cAMP elevation and downstream signaling pathways lead to a state where synapses are primed for potentiation. Interestingly, recent studies demonstrated that astrocytes increase cAMP signaling upon synaptic activity,^62^^,^^63^ an effect attributed to extracellular adenosine, which is independent of intracellular calcium,^63^ suggesting that our DdPAC-mediated interventions are recruiting an existing physiological synaptically activated pathway.

The comprehensive analysis of molecular changes induced by DdPAC stimulation sheds further light on the involvement of cAMP signaling in astrocytes in synaptic plasticity. Particularly, DdPAC stimulation in astrocytes involves the phosphorylation of proteins linked to the modulation of synapses and to the modulation of cell morphology. In fact, it is well known that cAMP contributes to morphological changes in astrocytes, as shown by forskolin-induced effects,^64^^,^^65^^,^^66^^,^^67^ noradrenaline and adrenaline activation in astrocyte cultures, and *in vivo* selective striatal astrocyte GPCR-Gi activation.^20^^,^^66^^,^^67^^,^^68^ Morphological changes in astrocytes have been associated with ARP2/3 activity,^69^ a major modulator of actin dynamics, which is also increased by DdPAC stimulation in our phosphoproteomic differential dataset. Moreover, DdPAC activation induced the phosphorylation of the PKA catalytic unit (PRKAR1A) and its effector proteins: adducin,^35^ PXN^36^ and VASP,^37^ highlighting the direct link between cAMP increases in astrocytes and the potential modulation of astrocyte morphology through actin and focal adhesion modulation. These effects are further supported by the induction of Rho GTPases signaling by DdPAC, which has been reported to modulate actin dynamics.^70^ In this line, a concerted neuronal activity and astrocyte-dependent morphology changes appear necessary for synaptic plasticity, as previously shown in studies on dendrite dynamics and motor behavior during critical periods.^7^

In addition, hemodynamic responses were induced *in vivo* by the stimulation of astrocytic DdPAC. This observation emphasizes the importance of incorporating the astrocytic cAMP signaling pathway as an additional mechanism in the broader framework of neurovascular coupling,^71^ while also demonstrating the ability of DWS to measure CBF *in vivo.*^40^ Interestingly, cAMP induced by PACs expressed in blood vessel cells has also been shown to induce local hemodynamic increases.^72^ Moreover, we observed increased AQP4 levels by immunofluorescence in postmortem tissue sections from the same WT mice. However, AQP4 protein levels were detected but not changed by DdPAC stimulation in our proteomic and phospho-proteomic datasets (data not shown). Therefore, the observed changes in AQP4 levels by immunofluorescence could be caused by other mechanisms, such as the increased expression of AQP4 in the plasma membrane. In this line, AQP4 membrane trafficking near glutamate synapse modulates synaptic transmission through astrocyte processes' motility.^47^ In turn, synaptic transmission involves the swelling of perisynaptic astrocyte processes as a consequence of extracellular K+ buffering and associated water movement through AQP4.^73^ Because synaptic activity is required for DdPAC-LTP induction, it could be that cAMP signaling in astrocytes contributes to synaptic plasticity by shaping astrocyte coverage of synapses and modulating synaptic and extra-synaptic glutamate and ion concentration through water mobilization, and therefore neuronal excitability, processes known to be altered in HD.^20^^,^^68^^,^^74^ Moreover, this aligns with the observed increase in GFAP expression in stimulated areas in the *in vivo* behavioral experiment, and increased the modulation of cytoskeletal processes in the (phospho)-proteomic study.

Notably, the selective cortical astrocytic cAMP induction *in vivo* was able to improve performance in the accelerated rotarod task, a known corticostriatal-dependent behavioral task.^51^^,^^52^ Indeed, alterations in astrocyte-neuron communication have been shown to impair motor learning, while sparing locomotion and motor coordination,^75^ similar to our results. However, divergent responses were observed by DdPAC stimulation in HD mice, with well-known alterations in astrocytic function^19^^,^^20^^,^^41^^,^^42^^,^^76^ and cAMP signaling.^15^^,^^16^^,^^20^^,^^43^^,^^77^ First, we observed that DdPAC stimulation in astrocytes induces higher brain hemodynamic responses. Moreover, it does not induce AQP4 increases in HD mice. At the behavioral level, DdPAC stimulation increased stereotypical grooming behavior and impaired coordination in HD mice. Thus, our results highlight the relevant role of astrocytes, and particularly the altered cAMP signaling in astrocytes, in HD pathophysiology.

Altogether, we provide compelling evidence of the multifaceted impact of cortical astrocytic cAMP signaling in synaptic potentiation, protein regulation, brain hemodynamic, and behavior. Moreover, our data demonstrated the potential of DdPAC as an excellent optogenetic tool to modulate cAMP signaling with red light, which might be used to reveal cell-type specific physiological and pathological roles of cAMP and could help to design novel therapeutic strategies.

### Limitations of the study

This study primarily focuses on the role of astrocytic cAMP in synaptic plasticity in wild-type mice, and hemodynamic and behavioral responses in the R6/1 mouse model of HD. However, the role of cAMP in synaptic plasticity in HD mice is not known, and future studies should explore how DdPAC stimulation shapes synaptic plasticity also in the R6/1 mouse model to provide mechanistic insights in healthy and disease conditions. Furthermore, while the effects on synaptic plasticity are blocked by PKA blockers, and proteomic and phosphoproteomic data show PKA and downstream signaling modulation, direct cAMP measurements are needed to establish causal links between DdPAC activation effects and cAMP.

## Resource availability

### Lead contact

Further information request should be directed to H. Rheinallt Parri (h.r.parri@aston.ac.uk) and/or Mercè Masana (mmasana@ub.edu).

### Materials availability

DdPAC and EGFP constructs will be provided to Addgene.

### Data and code availability

Mass spectrometry data and search results files were deposited in the Proteome Xchange Consortium/PRIDE with the identifiers PXD054633 (Proteomics data) and PXD054635 (phospho-proteomics data).

## Acknowledgments

This research is part of the NEUROPA and GlioLight project. The NEUROPA Project has received funding from the European Union’s Horizon 2020 Research and Innovation Program under Grant Agreement No. 863214. The GlioLight project has received funding from the European Innovation Council under grant Agreement 101129705. The Aston Institute for Membrane Excellence (AIME) is funded by UKRI’s Research England as part of their Expanding Excellence in England (E3) fund. MK is funded by Horizon Europe Marie Skłodowska-Curie Actions (101104889). This study was supported by grants from the Ministerio de Ciencia y Innovación (Spain) y la Agencia Estatal de Investigación, under projects CNS2023-143999 (MM.), PID2021-124896OA-I00 (M.M.), and no. PID2020-119386RB-I00 (J.A. and M.J.R.); Instituto de Salud Carlos III, Ministerio de Ciencia, Innovación y Universidades and European Regional Development Fund (ERDF) [CIBERNED, to J.A.], Spain. Also, the project has been supported by María de Maeztu Unit of Excellence (CEX2021-001159), the Institute of Neurosciences of the University of Barcelona, Ministry of Science, Innovation, and Universities, and the Agència de Gestió d'Ajuts Universitaris i de Recerca (AGAUR, Catalunya) (2021SGR01086).

We thank Ju Chen (UCSD) and Alfonso Araque (University of Minnesota) for the IP_3_R2^-/-^ mice. The authors acknowledge the support of Aston University Biomedical Facility for the purpose of providing infrastructure support within the College of Health and Life Sciences. We are also grateful to the staff of the Confocal Microscopy Service and the Animal Experimental Unit of the Scientific and Technological Centers of the University of Barcelona (CCiTUB) and to Maria Teresa Muñoz, Ana Maria Lopez, and Silvia Artigas for excellent technical support.

## Author contributions

LSR, NMN, SS, ER, MJR, JA, DD, AM, AB, IM, HRP, and MM designed the experiments. LSR, NMN, EZ, MK, MG, VB, FD, EA-C, ES, JF, SCB, AC, AB, MM performed experiments. LSR, NMN, EZ, ES, JF, MJR, AB, HRP, and MM analyzed data. SS, ER, MJR, JA, DD; AM, AB, IM, HRP, MM obtained funding. LSR, NMG, AB, IM, DD, AM, HRP, and MM wrote the first article draft. All authors contributed to the article review and accepted the final draft.

## Declaration of interests

The authors declare no competing interests.

## Declaration of generative AI and AI-assisted technologies in the writing process

During the preparation of this work, the author(s) used ChatGPT and Copilot in order to correct grammar and improve clarity of already written text.

## STAR★Methods

### Key resources table

**Table:** 

REAGENT or RESOURCE	SOURCE	IDENTIFIER
**Antibodies**		

Chicken monoclonal/polyclonal anti-GFP	Synaptic systems	132 006; RRID:AB_2713983
Rabbit monoclonal/polyclonal anti-GFAP	Agilent	z0334; RRID: AB_10013382
Rabbit monoclonal/polyclonal anti-AQP4	Merck	AB3594; AB_91530
Cy3 555 anti-rabbit IgG	Jackson Immunoresearch	JAC 111-165-003; RRID:AB_2338000
Rabbit anti-mCherry	Abcam	#ab183628; RRID:AB_2650480
Mouse anti-NeuN	Merck	#MAB377; RRID:AB_2298772
Mouse anti-mCherry	Abcam	#ab125096; RRID:AB_11133266
AlexaFluorTM 647 anti-mouse	Invitrogen	A21236; RRID:AB_2535805
AlexaFluorTM 488 anti-chicken	Invitrogen	A11039; RRID: AB_2534096
Alexa Fluor 568 anti-rabbit	Molecular probes	#A-11011; RRID: AB_143157
Alexa Fluor 660 anti-mouse	Molecular probes	#A- 21055; RRID: AB_2535722
Alexa Fluor 488 anti-mouse	Abcam	#ab150113; RRID: AB_2576208
Alexa Fluor 568	Molecular probes	#A-11011; RRID: AB_143157

**Bacterial and virus strains**		

AAV9-GFAP-DdPAC-3xFLAG-WPRE	This paper	N/A
AAV9-GFAP-eGFP-WPRE	This paper	N/A
pAAV.hSyn.iGluSnFr.WPRE.SV40 (AAV1)	Gift from Loren Looger. https://www.addgene.org/browse/article/6319/	Addgene viral prep # 98929-AAV1; RRID:Addgene_98929
AAV5-GfaABC1D-mCherry-hPMCA2w/b	https://www.addgene.org/browse/article/28193459/	Addgene #111568; RRID: Addgene_111568
AAV1-CaMKIIa-hChR2(H134H)-eYFP-WPRE.hGH	–	Addgene#26969-AAV1; RRID:Addgene_26969

**Chemicals, peptides, and recombinant proteins**		

Protease inhibitor cOmplete Mini	Roche	#11836153001
Phosphatase inhibitor PhosSTOP	Roche	#4906845001
PKA inhibitor KT5720	Hello Bio: https://hellobio.com/kt-5720.html	HB0361
5, 7, DCK	Biotechne: https://www.bio-techne.com/p/small-molecules-peptides/5-7-dichlorokynurenic-acid-sodium-salt_3698	3698
MK801	Hello Bio: https://hellobio.com/mk801.html	HB0004
D-AP5	https://hellobio.com/catalogsearch/result/?q=AP5	HB0225

**Critical commercial assays**		

SYBR Green	Thermo Fischer Scientific	4309155
Bradford assay kit	BioRad	–
Pierce™ Peptide Desalting Spin Columns	Thermofisher	–
High-Select™ TiO2 Phosphopeptide enrichment Kit	ThermoFisher	–

**Deposited data**		

Raw proteomic data	This paper	Proteome Xchange Consortium/PRIDE: PXD054633
Raw phospho-proteomic data	This paper	Proteome Xchange Consortium/PRIDE: PXD054635

**Experimental models: Organisms/strains**		

Mouse: C57BL/6	Charles River	Cat#027; RRID:IMSR_JAX:000664
Mouse: IP3R2−/− (C57BL/6 background)	Prof. Araque (Cajal Institute, Madrid), with permission from Prof. Ju Chen (UC San Diego)	N/A
Mouse: R6/1 HD model: B6CBA-Tg(HDexon1) 61Gpb/1J	The Jackson laboratory	RRID:IMSR_JAX:002809

**Software and algorithms**		

Spectronaut	Biognosys	–
ImageJ	Schindelin et al.^78^	https://imagej.nih.gov/ij/; RRID: SCR_003070
MATLAB version 9.4.0.813654 (R2018a)	MATLAB Works	https://es.mathworks.com/products/matlab.html; RRID:SCR_001622
SMART 3.0 software	Panlab	https://www.panlab.com/es/productos/smart-video-tracking-software-panlab; RRID:SCR_002852
Graphpad Prism version 10.0.0	Prism	https://www.graphpad.com/; RRID:SCR_002798

**Other**		

780 nm LED Fiber Light source	Thorlabs: https://www.thorlabs.com/thorproduct.cfm?partnumber=M780F2	MT780F2
660 nm LED Fiber Light source	Thorlabs: https://www.thorlabs.com/thorproduct.cfm?partnumber=M660FP1	M660FP1
685 nm single LD Fiber Light Source (40 mW)	Doric Lenses	D470-0060
473 nm diode-pumped solid-state blue laser	Laserglow	R471005GX
Diffusion Correlation Spectroscopy system	This paper	N/A

### Experimental model and study participant details

#### Animals

All animal procedures were approved by local ethical review at Aston University or the Animal Experimentation Ethics Committee of the Universitat de Barcelona (356/19) and Generalitat de Catalunya (11070) and performed in accordance with the United Kingdom Animals Scientific Procedures Act of 1986 (PPL-PA7B2DD3C), or Spanish RD 53/2013 and current EU legislation, respectively. The 3Rs, replacement, refinement and reduction were considered for planning all animal procedures.

C57BL/6J mice (Charles River) were used for MEA recordings (between P28-P60 age mice, both males and females) and omics analyses (between 8 and 12 weeks of age, females). For MEA, IP_3_R2^−/−^ mice were also used. IP_3_R2-/2 mice were imported from the laboratory of Prof. Araque (Cajal Institute, Madrid), with the permission of their creator, Prof. Ju Chen (San Diego), re-derived into the C57Bl/6J background and amplified by crossing homozygotes. IP_3_R2-/2 mice genotyping was conducted by Transnetyx (TN, United States).

DWI and behavioral experiments were performed using symptomatic R6/1 transgenic mouse (B6CBA background), expressing the exon-1 of mutant huntingtin with ∼145 CAG repeats at the time of experiments (from 8 to 18 weeks, both males and females). Genotypes were obtained by polymerase chain reaction (PCR) from ear biopsy and WT littermates were used as the control group. All animals were group-housed in a room kept at 19°C–22°C and 40–60% humidity under 12:12h light/dark cycle.

### Method details

#### Adeno-associated virus constructs

The recombinant adeno-associated virus (AAV) 9 vectors AAV9-GFAP-DdPAC-3xFLAG-WPRE (AAV-GFAP-DdPAC) and AAV9-GFAP-eGFP-WPRE (AAV-GFAP-GFP) were produced following the co-transfection method as described previously and purified through iodixanol gradient ultracentrifugation.^79^ Concentration and buffer exchange were carried out using PBS containing 0.001% Pluronic. The titers of AAV vector stocks were subsequently determined using the real-time quantitative PCR titration method^80^ with SYBR Green (Thermo Fischer Scientific) and were ∼1-3x10^14^ vg/ml.

pAAV.hSyn.iGluSnFr.WPRE.SV40 (AAV1) was a gift from Loren Looger (Addgene viral prep # 98929-AAV1). AAV5-GfaABC1D-mCherry-hPMCA2w/b (CalEx) (Addgene #111568) and AAV1 containing Channelrhodopsin (ChR2) under CaMKII promoter (AAV1-CaMKIIa-hChR2(H134H)-eYFP-WPRE.hGH, AAV-CHR2, titer ∼1-3x10^12^) (#26969-AAV1, Addgene) were also used.

#### Stereotaxic surgeries

Stereotaxic surgeries were performed 4 weeks before the start of the stimulation. The following viruses were injected for different purposes: a) For multielectrode array recordings, AAV-GFAP-DdPAC was injected into the somatosensory cortex of WT mice, along with AAV-GFAP-GFP to visualize the stimulation area. In some experiments, CalEx mouse was injected. b) For omics experiments, AAV-GFAP-DdPAC was injected bilaterally into the M2 cortex of WT mice, with control animals receiving AAV-GFAP-GFP. c) For diffuse wave spectroscopy imaging, AAV-GFAP-DdPAC and AAV-CHR2 were injected into the somatosensory cortex of WT and R6/1 mice. d) For *in vivo* DdPAC stimulation and behavior, AAV-GFAP-DdPAC and AAV-GFAP-GFP were injected in the M2 cortex of WT and R6/1 mice.

Surgeries were performed as previously described.^24^^,^^49^^,^^81^ Briefly, isoflurane anesthesia (5% induction, and 1.5% maintenance) was used and Meloxicam (2 mg/kg s.c.) was injected before the surgery to avoid pain and reduce inflammation. A volume of 0.5–1 μL of corresponding viral constructs was injected at the following coordinates for each of the experiments, taking bregma and dura mater as a reference: a) For multielectrode array experiments: AP 1.5, ML -3.5, DV 0.5. b) For omics experiments: AP 2.46, ML±1, DV +0.6. c) For diffuse wave spectroscopy imaging: AP -0.2, ML -1.8, DV -0.4. d) For DdPAC *in vivo* stimulation and behavior: AP 2.46, ML±1, DV +0.6. For DdPAC stimulation *in vivo*, additional fiber-optic cannulas (1 mm length, 400 μM diameter) were implanted bilaterally on the M2 cortex (AP 2.46, ML±1, DV +0.6) from bregma and dura matter during surgery and secured using dental cement.

#### DdPAC stimulation

For *ex vivo* recordings, experiments were performed in a complete light-proof environment. Baseline acquisitions were done under a 780 nm single LED Fiber Light source (Thorlabs) directed exactly above the acute cortical slice for 20 min to enable complete deactivation of DdPAC construct before application of a 660 nm LED (Thorlabs) under manual control.

For *in vivo* stimulations, red-light from a 685 nm single LD Fiber Light Source (Doric Lenses) was delivered at ∼5–18 mW (measured at the end of the patch cord) and was controlled by the free Doric Neuroscience studio Software. For omics, zirconia sleeves were used to connect the patch cord to the fiber-optic cannulas and stimulation was performed in freely moving animals for 10 min (1 s 685 nm ON; 4 s 685 nm OFF). For behavioral studies, stimulation was performed for 5 min in each hemisphere for 4 days (1 day every 3 days). For diffuse wave spectroscopy imaging, stimulation was performed in anesthetized head-fixed mice. The fiber-optic patch cord was placed above the surface of the dura-matter, after drilling a hole the size of the tip of the cannula on the skull. For ChR2 stimulation, a 473 nm laser (Laserglow) was applied at 10 Hz with 5 ms pulses and 10 mW power for 1 min.

#### Slice preparation for multielectrode array recordings

Slices of the mouse barrel cortex were prepared as previously described.^24^^,^^78^ Briefly, mice were anesthetized with isoflurane overdose (5%) followed by cervical dislocation and the whole brain was removed and placed in an ice-cold bathing solution containing the following (in mM); NaCl 120, NaHCO3 25, KCl 1, KH2PO4 1.25, MgSO4 5, CaCl2 2, and glucose 10 and pH 7.2. Brains were glued to a metal block using cyanoacrylate adhesive and submerged in the bath of a Campden 7000 vibroslicer (Campden Instruments) containing the same bathing solution maintained at <5°C and bubbled with 95% O2 and 5% CO2. Cortical slices (350 μm) were cut in the coronal plane and maintained in the bathing solution at room temperature oxygenated (95% O2/5% CO2) for at least 1 h recovery period before use. Recordings were performed in an artificial CSF (aCSF) solution containing the following (in mM): NaCl 120, NaHCO3 25, KCl 2, KH2PO4 1.25, MgSO4 1, CaCl2 2 and glucose 10.

#### Multielectrode array recordings

The MED64 system (Alpha MED Scientific, Japan) with MEA probes consisting of 64 platinum electrodes, each 50 μm in diameter and an inter-electrode distance of 150 μm in an 8 × 8 arrangement pattern was used. Probes were pre-coated with 0.1% w/v polyethyleneimine (PEI) in a borate buffer overnight (>12 h) at RT before first use. Cortical slices were carefully placed on the probes using an inverted microscope (10-X objective lens) such that the somatosensory barrels were on identified electrodes to enable selective stimulation of L4. fEPSPs were recorded from L2/3. Slices were held in position with mesh and slice harp. Once in position, a period of 15 min was allowed following aCSF perfusion (2 mL/min) to allow for equilibration before recordings were made. Synaptic stimulation was achieved by delivering 0.2 ms biphasic pulses to one electrode laying beneath an L4 region and fEPSPs were recorded on electrodes laying beneath L2/L3 somatosensory cortex. The stimulating intensity was set at 50% of a maximal fEPSP rising slope determined from the I/O curve produced using different stimulating intensities for each slice. A relatively low stimulating frequency [0.008 Hz] was used to evoke fEPSPs to avoid potential continuous stimulation-related effects on slice responses. Baseline recordings were done for 20 min before either stimulation with 660 nm light and/or TBS protocol and plasticity was assessed 1 h post-induction.

#### Live confocal imaging

Live confocal imaging was carried out using a Zeiss Axiovert 200M epifluorescent microscope using Leica Application Suite Advanced Fluorescence (LASAF) microscopy software (Leica Microsystems, Milton Keynes, UK). Acute cortical slices transfected with both hSyn.iGluSnFr and AAV-GFAP-DdPAC constructs were continuously perfused with aCSF and illuminated with 460 nm which is optimum for iGluSnFR stimulation in the green emission channel.^82^ Experiments were done in the presence of 50 μL DL-*threo*-beta-benzyloxyaspartate (DL-TBOA) (Tocris) to block excitatory amino acid acid transporters. Images for iGluSnFR signals were collected in a time-lapse mode in 1 s intervals before, during and after evoked glutamate release. For a positive control, some experiments involved bath application of glutamate (100 μM) to acute slices co-injected with both constructs. To assess DdPAC’s effect on glutamate increment, experiments were done in the dark and acute slices were illuminated with 660 nm light following collection of baseline measurements. The iGluSnFR signal was expressed as the ΔF/F0, = (F (t)- F0)/F0 where F(t) stands for intensity over time, and F0 is the baseline intensity averaged over ∼60 s prior to the stimulus.

#### Proteomics and phosphoproteomics

##### Tissue obtention

Animals were euthanized immediately after 10 min of light stimulation. The tissue surrounding the cannula including the frontal cortex was obtained from both hemispheres. The tissue obtained from the left hemisphere was used for proteomics and phosphoproteomics analyses.

##### Sample preparation

Tissue samples were homogenized in a lysis buffer (7 M urea, 2 M thiourea, 50 mM dithiothreitol (DTT), supplemented with protease (cOmplete Mini, Roche #11836153001) and phosphatase inhibitors (PhosSTOP, Roche #4906845001). Lysates were centrifuged at 20,000 g (1 h, 15 °C), and the resulting supernatant was quantified with the Bradford assay kit (BioRad, Barcelona, Spain). To obtain the phosphorylated peptide sample fraction, 600 μg of protein was separated for protein digestion. Proteins were reduced with DTT (final concentration of 20 mM; room temperature, 30 min), alkylated with iodoacetamide (final concentration of 30 mM; room temperature, 30 min in the dark), diluted to 0.9 M with ABC and digested with trypsin (Promega, Madison, WI, USA; 1:20 w/w enzyme protein ratio, 18 h, 37 °C). Protein digestion was interrupted by acidification (pH < 6, acetic acid), and the resulting peptides were cleaned up using Pierce Peptide Desalting Spin Columns (ThermoFisher Sci., Waltham MA, USA). The following phosphorylated peptide enrichment was performed using the High-Select TiO2 Phosphopeptide enrichment Kit (ThermoFisher Sci., Waltham, MA, USA) according to the manufacturer’s instructions. Finally, the enriched phosphopeptide sample fraction was cleaned-up as described before and dried down in a Speed-Vac system. A 10 μg aliquot of cleaned-up peptides from protein digestion was set aside for total protein analyses.

##### Data independent acquisition mass spectrometry

Dried-down peptide samples were reconstituted with 2% ACN-0.1% FA (Acetonitrile-Formic acid), spiked with internal retention time peptide standards (iRT, Biognosys), and quantified by NanoDropTM spectrophometer (ThermoFisher Sci.) prior to LC-MS/MS analysis using an EASY-1000 nanoLC system coupled to an EZ-Exploris 480 mass spectrometer (Thermo Fisher Sci.). Peptides were resolved using C18 Aurora column (75 μm × 25cm, 1.6 μm particles; IonOpticks) at a flow rate of 300 nL/min using a 60-min gradient (50 °C): 2%–5% B in 1 min, 5%–20% B in 48 min, 20%–32% B in 12 min, and 32%–95% B in 1 min (A = FA, 0.1%; B = 100% ACN:0.1% FA). Peptides were ionized using 1.6 kV spray voltage at a capillary temperature of 275 °C. Sample data were acquired in data-independent acquisition (DIA) mode with full MS scans (scan range: 400 to 900 m/z; resolution: 60,000; maximum injection time: 22 ms; normalized AGC target: 300%) and 24 periodical MS/MS segments applying 20 Th isolation windows (0.5 Th overlap: Resolution: 15000; maximum injection time: 22 ms; normalized AGC target: 100%). Peptides were fragmented using a normalized HCD collision energy of 30%.

##### Bioinformatics and statistical analysis

Mass spectrometry data files were analyzed using Spectronaut (Biognosys) by direct DIA analysis (dDIA). MS/MS spectra were searched against the Uniprot proteome reference from the mouse database using standard settings. The enzyme was set to trypsin in a specific mode. On the one hand, Carbamidomethyl (C) was set as a fixed modification, and oxidation (M), acetyl (protein N-term), deamidation (N), and Gln-> pyro-Glu as variable modifications for total protein analysis. On the other hand, Carbamidomethyl (C) was set as a fixed modification, and oxidation (M), acetyl (protein N-term), and Phospho (STY) as variable modifications for phospho-proteome analysis. Identifications were filtered by a 1% Q-value.

The obtained quantitative data for total protein were exported to Perseus software (version 1.6.15.0)^83^ for statistical analysis and data visualization. For total protein analysis, unpaired Student’s t test was used for direct comparisons. Statistical significance was set at *p*-value lower than 0.05 in all cases and 1% peptide FDR threshold was considered. Differentially expressed proteins were considered significant when their absolute fold change was below 0.77 (downregulated proteins) and above 1.3 (up-regulated proteins) on a linear scale. Quantitative data obtained from the phosphoproteome were collapsed using a custom coded plugin Peptide Collapse (v.1.4.4) in Perseus (v.1.6.15.0) that convert a normal Spectronaut report into a site-level report.^84^ Plugin settings were set as default grouping posttranslational modifications (PTMs) by sample (FileName), collapsing matrix by site-level and setting the PTM localization probabilities filter at more than 0.75. Statistical analyses were conducted following the same protocol as the total protein study.

The functionality associated with the differential (phospho)proteomes was assessed using Metascape^85^ using default settings (min. overlap: 3, min. enrichment: |1.5|, *p* < 0.05). Synaptic ontologies were obtained from the SYNGO tool.^86^ Protein interactomes and activation predictions were analyzed using QIAGEN’s Ingenuity Pathway Analysis (IPA; QIAGEN Redwood City).^87^

#### DCS-based brain hemodynamics imaging system

Diffusion Correlation Spectroscopy (DCS)^40^ was used to measure blood flow in the cortex of mice brains. In DCS, a coherent laser light illuminates the tissue, and the diffusely reflected light scattered within the tissue is analyzed (Figure 4A). The motion of red blood cells within the tissue causes fluctuations in the intensity of the scattered light. These temporal intensity fluctuations are captured by a high-speed camera. The basic principle of DCS is that the faster the red blood cells move, the shorter the correlation time will be. The time correlation, derived from the auto-correlation function of the temporal intensity fluctuations, can be used to assess cerebral blood flow in brain tissues.

Our setup for the transcranial blood flow imaging comprises a high-speed camera (EoSens 3CL, Germany), a frame grabber card (Silicon Software, Germany), an 850 nm distributed Bragg reflector single-frequency NIR laser (Thorlabs Inc, USA), and a collimator with a diffuser. At the surface of the studied object, the laser intensity is maintained at 20 mW/cm^2^. Crossed polarizers are employed on the collimator and camera objective to eliminate surface reflections. This configuration enables the reconstruction of blood flow images with a resolution of 240 x 240 pixels and facilitates the calculation of the temporal intensity autocorrelation function of light at each of the measured pixels within a range of 10ˆ-4 to 1 s. Specifically, in our study, we recorded 50,000 frames per measurement at a frame rate of 8,000 fps for subsequent processing.

Blood flow index (BFI) maps were calculated as an average value of the power spectrum obtained through the Fourie transform of the measured autocorrelation function in each pixel according to the equation.^88^

$$BFI_{i,j}=\left(\int \omega S\left[g_{2}^{i,j}\left(t\right)-1\right]d\omega \right)/\left\langle I_{i,j}\right\rangle$$

Here $S\left[g_{2}\left(t\right)-1\right]$ is the power spectrum obtained through the Fourier transform of the temporal autocorrelation function, ⟨*I*⟩ is the average DC signal from the pixel, *i* and *j* are correspondingly the row and column number of the image map identifying the pixel. All data were processed on MATLAB (version 9.4.0.813654 (R2018a), and final images were opened with ImageJ software,^89^ mounted as stack images and intensity of the whole hemisphere was measured using the measure tool plugin in all images obtained from each mouse.

#### Behavioral assessment

All behavioral tests were performed by an experimented observer during the light phase and animals were habituated to the experimental room for at least 1 h before testing. All testing apparatus were carefully cleaned with water and dried between tests and animals.

##### Open field

Spontaneous locomotor activity and exploratory behavior were assessed in an open field at 14 weeks of age.^49^^,^^50^ The test was performed using a gray square arena (40 × 40 × 30 cm3) with dim light (∼20 lx). Mice were left in the center of the apparatus and allowed to explore the arena for 15 min. Animals were tracked and recorded with SMART 3.0 software (Panlab). The number of events and time spent doing rearing and grooming were measured to assess exploratory and stereotypic behavior.

##### Accelerating rotarod

Motor learning and coordination were evaluated in an accelerating rotarod.^49^^,^^50^ Mice were placed on a motorized rod (30 mm diameter) with a rotation speed gradually increased from 4 to 40 rpm over 5 min and the latency to fall was recorded to assess motor learning. The procedure was performed four times per day with a 1-h inter-trial interval for 3 consecutive days, 12 trials in total.

##### Balance beam

Motor coordination and balance were evaluated by measuring the ability of the mice to move along a narrow beam without falling or slipping.^49^^,^^50^ The beam consisted of a wooden square bar (50 cm long with 1.3 cm face), divided by 5 cm-frames and placed horizontally 50 cm above the bench surface, with each end mounted on a support. Animals could walk for 2 min along the beam to familiarize themselves with the set-up. 4 h later, the procedure was repeated and the number of frames crossed and slips were measured.

##### Vertical pole

Motor coordination was evaluated using a vertical pole.^50^ Animals were placed on top of a vertical 35 cm long bar with a diameter of 1 cm. Mice were positioned grasping the pole with four paws and the head pointing upwards. A training was performed 3 times per day for 2 consecutive days. Testing was performed on the third day and the time latency of the mouse to turn downwards and completely descend the pole was recorded.

#### Immunofluorescence and imaging acquisition

For DdPAC expression validation in WT and HD brains, mice were sacrificed by cervical dislocation and brains were post-fixed with 4% PFA and dehydrated in a PBS/sucrose gradient [from 15% (48h postmortem) to 30% (72 h postmortem)] with 0.02% sodium azide and kept at 4°C. Sections (30 μm) were cut on a vibratome (Leica VT1000S) and cryopreserved (30 mL Ethylene glycol; 30 mL Glycerol; 25 mL TB (1M Tris HCl pH 7,5); 15 mL H2O miliQ for 1 L of solution) at −20°C. Free-floating sections were first washed in PBS 0.01M, then treated with 50 mM NH4Cl, permeabilized with 0.01M PBS containing 0.5% Triton X-100 and blocked for 2 h with a solution of 0.01M PBS with 0.02% azide, 0.3% Triton X-100, 0.2% BSA and 5% normal goat serum (Pierce Biotechnology). Permeabilization and blocking duration was exceptionally combined and extended to overnight incubation in the blocking solution for the staining of 300 μm sections obtained from MEA. Sections were then incubated overnight with anti-GFP chicken (132 006, Synaptic Systems) or anti-flag mouse (F1804, Sigma Aldrich) to evaluate the expression of AAV-GFAP-GFP and AAV-GFAP-DdPAC viruses, respectively. Anti-GFAP rabbit (z0334, DAKO) and anti-AQP4 rabbit (AB3594, Merck) antibodies were used to assess GFAP-positive astrocytes. Primary antibodies were diluted in a solution of 0.01M PBS with azide 0.02%, 0.5% Triton X-100, BSA 0.2% and 5% normal goat serum. After washing with 0.01M PBS, brain sections were incubated for 1:30 h with Cy3 555 goat-anti rabbit IgG (JAC 111-165-003), AlexaFluorTM 647 anti-mouse (A21236, Invitrogen), AlexaFluorTM 488 anti-chicken (A11039, Invitrogen), diluted in 0.01% PBS. After secondary antibody incubation, sections were washed in 0.01M PBS and mounted on microscope slides using DAPI Fluoromount-G (Southern Biotechnology) and kept in the dark. All washes and incubations were completed on a shaker and at RT, except for the primary antibody incubation, which was conducted at 4 °C. Fluorescence images were acquired by an epifluorescence microscope (DMI6000 Widefield Leica) and a confocal microscope (Carl Zeiss LSM880). Mosaic images to identify the virus-infected zone were acquired with 10× objective lenses, except sections obtained from MEA which were taken with Carl Zeiss LSM880 40× objective. Immunofluorescence staining for AQP4 was performed simultaneously on all samples to ensure consistency. Images were acquired using identical microscope settings, including laser intensity, exposure time, and gain. Similarly, GFAP immunofluorescence staining from the behavioral experiment was conducted on all samples in parallel, and image acquisition was performed under the same lighting parameters. GFAP and AQP4 fluorescence intensity was calculated using ImageJ/Fiji software.^89^

For IP3R2-/2 immunofluorescence, brains were post-fixed for 3 h in 4% PFA and placed in 10 mM PBS with 0.1% sodium azide and 30% sucrose for cryoprotection and long-term storage. Free-floating 30 μm coronal sections were prepared using a Leica vibratome (Leica Microsystems Inc., USA). The slices were transferred to well-plates and washed three times for 10 min with 500 μL PBS-T (0.1% Triton X- 100 in sterile 10 mM PBS) on a circular rocker. To prevent non-specific binding, the slices were incubated for 1 h in 250 μL of freshly prepared blocking solution (4% bovine serum albumin, 3% donkey serum and 0.1% Triton X-100 in sterile 10 mM PBS) per well. Primary antibodies were prepared using a 1:1 solution of blocking solution and PBS-T. 250 μL of the antibody solution was added to each well and the slices were incubated overnight, at 4°C. The samples were washed twice with 500 μL 10 mM PBS for 10 min, once with 500 μL 10 mM PBS for 20 min and twice with 500 μL PBS-T for 10 min. Secondary antibodies were prepared using a freshly prepared 1:1 solution of blocking solution and PBS-T. 250 μL of the antibody solution was added to each well and the samples were incubated for 1 h at room temperature on a circular rocker, in dark conditions. Next, the samples were immersed in 250 μL of DAPI (5 μg/mL, in 10 mM PBS) for 10 min, and washed twice with 500 μL PBS-T and once with 500 μL 10 mM PBS for 5 min. The slices were mounted on glass microscope slides using Molecular Probes SlowFade mountant and allowed to dry overnight at room temperature. Imaging was performed with a Zeiss LSM 880 upright confocal microscope. The following combinations of primary and secondary antibodies were used. To rule out hPMCA2w/b pump expression in neurons: rabbit anti-mCherry (Abcam #ab183628, diluted 1:500) with rabbit Alexa Fluor 568 (Molecular Probes #A-11011, diluted 1:500), and mouse anti-NeuN (Merck #MAB377, diluted 1:500) with mouse Alexa Fluor 660 (Molecular Probes #A- 21055, diluted 1:500). To verify hPMCA2w/b pump expression in astrocytes: mouse anti-mCherry (Abcam #ab125096, diluted 1:500) with mouse Alexa Fluor 488 (Abcam #ab150113, diluted 1:500), and rabbit anti-GFAP (Agilent #Z0334, diluted 1:500) with rabbit Alexa Fluor 568 (Molecular Probes #A-11011, diluted 1:500). DAPI was used in both preparations to locate the cell body of astrocytes or neurons. Unless otherwise specified, all reagents were purchased from MilliporeSigma, Inc., Germany. For cell staining colocalization a 200 μm × 200 μm ROI was taken per slice. mCherry (CalEX) and astrocyte (GFAP) colocalization analysis showed 90.4% colocalization of mCherry in GFAP positive cells (*n* = 94 mCherry positive cells, 3 animals, 4 slices per animal). Analysis of neuronal expression showed no co-localisation of mCherry with NeuN positive cells (*n* = 304 neuN positive cells, 3 animals, 4 slices per animal).

### Quantification and statistical analysis

Statistical analyses were performed on GraphPad Prism version 10.0.0 Software. Data are expressed as mean ± SEM and *p* values <0.05 were considered significant (*p* < 0.05 (∗), *p* < 0.01 (∗∗), and *p* < 0.001 (∗∗∗). For comparison between LTP and baseline in MEA recordings, and glutamate fluorescence before and after induction, paired Student’s t test was used. Differences between two independent groups were determined by Mann-Whitney test. For hemodynamic. immunofluorescence and behavioral experiments, the one-sample T-test, two-tailed unpaired Student’s t test, two-way or three-way ANOVA, followed by Bonferroni post hoc test were used when appropriate. Statistics are indicated in results and/or Figure legends.

### Additional resources

All data are available in the main text or the supplementary materials. Mass spectrometry data and search results files were deposited in the Proteome Xchange Consortium/PRIDE with the identifiers PXD054633 (Proteomics data)) and PXD054635 (phosphoproteomics data). DdPAC constructs will be provided to Addgene.
