# Supplementary material for: Photoactivated adenylyl cyclase in cortical astrocytes promotes synaptic potentiation and reveals alterations in Huntington’s disease
Source: iScience. 2025 Sep 24;28(11):113640. doi: 10.1016/j.isci.2025.113640 (PMC12554215; doi:10.1016/j.isci.2025.113640)
Supplement: Document S1. Figures S1–S3 [file mmc1.pdf]

iScience, Volume *28*

Supplemental information

Photoactivated adenylyl cyclase in cortical astrocytes promotes synaptic potentiation and reveals alterations in Huntington's disease

Laia Sitjà-Roqueta, Neville M. Ngum, Evgenii A. Zherebtsov, Melike Küçükerden, Maryam Givehchi, Valentina Bova, Francis Delicata, Elena Anaya-Cubero, Enrique Santamaria, Joaquín Fernández-Irigoyen, Sara Conde-Berriozabal, Anna Castañé, Sergei Sokolovski, Edik Rafailov, Manuel J. Rodríguez, Jordi Alberch, Deniz Dalkara, Andreas Möglich, Alexander Bykov, Igor Meglinski, H. Rheinallt Parri, and Mercè Masana

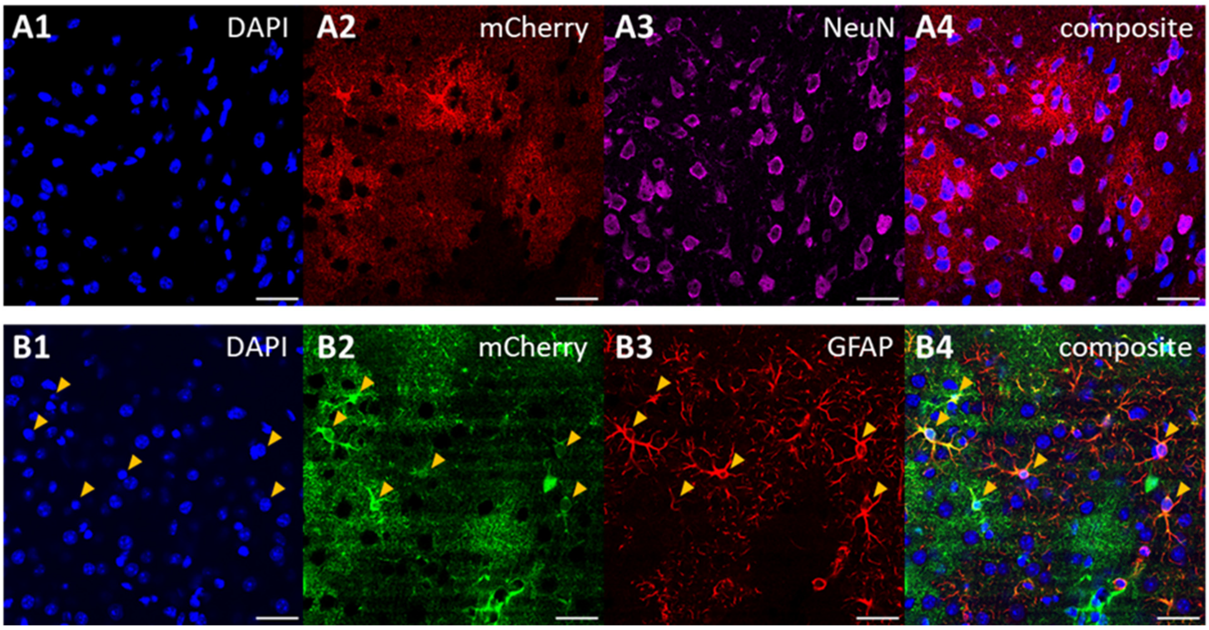

SUPLEMMENTARY FIGURE 1. Astrocyte-selective expression of AAV5-GfaABC1D-mCherry-hPMCA2w/b pump (CalEx) in mouse somatosensory cortex. A1-4. Immunohistochemistry with **(A1)** DAPI, **(A2)** anti-mCherry, **(A3)** anti-NeuN antibodies and **(A4)** overlap of the three channels shows lack of calEx expression in neurons, as suggested by the lack of colocalization of mCherry and NeuN labelling (Scale bar: 30 µm, 3 animals, 4 slices per animal were analyzed). **(B1-4)** Immunohistochemistry with **(B1)** DAPI, **(B2)** anti-mCherry, **(B3)** anti-GFAP antibodies and **(B4)** overlap of the three channels shows that the hPMCA2w/b pump was expressed in astrocytes, Arrows indicate astrocyte somata with colocalized mCherry and GFAP labelling (Scale bar: 30 µm, 3 animals, 4 slices per animal were analyzed).

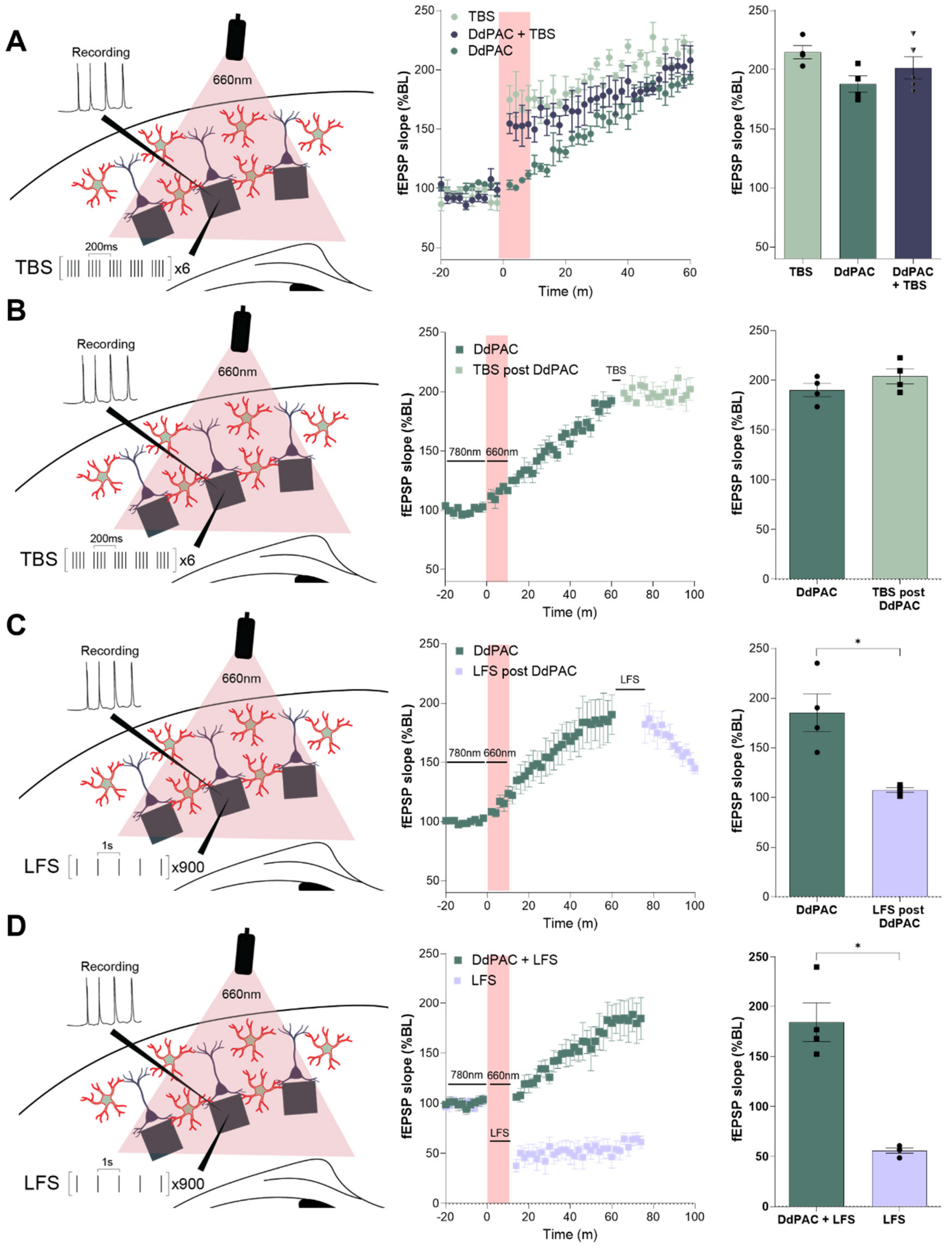

SUPPLEMENTARY FIGURE 2. DdPAC activation instigates an activity dependent but frequency independent potentiation. A-D. Left panels show schematic representation of the experimental setup. Middle panels show the induction of fEPSP slope. Right panels show fEPSP slope. **A**. Combination of TBS with DdPAC stimulation with 660 nm light. **B**. Effects of TBS stimulation after DdPAC stimulation with 660 nm light. **C**. Effects of LFS stimulation after DdPAC

stimulation with 660 nm light. **D**. Combination of LFS with DdPAC stimulation with 660 nm light. For **A-D**, Middle panels show plots of fEPSP slope at experimental time points where symbols are mean ± SEM for experiments in separate slices (n=4-5), with symbols corresponding to indicated experimental conditions. Panels on right show summary bar graphs of mean ± SEM (n=4-5) of normalised fEPSP slope 60 minutes post stimulation where each point represents data from a single slice. Significant differences between groups were determined by Mann-Whitney test (* $p < 0.05$).

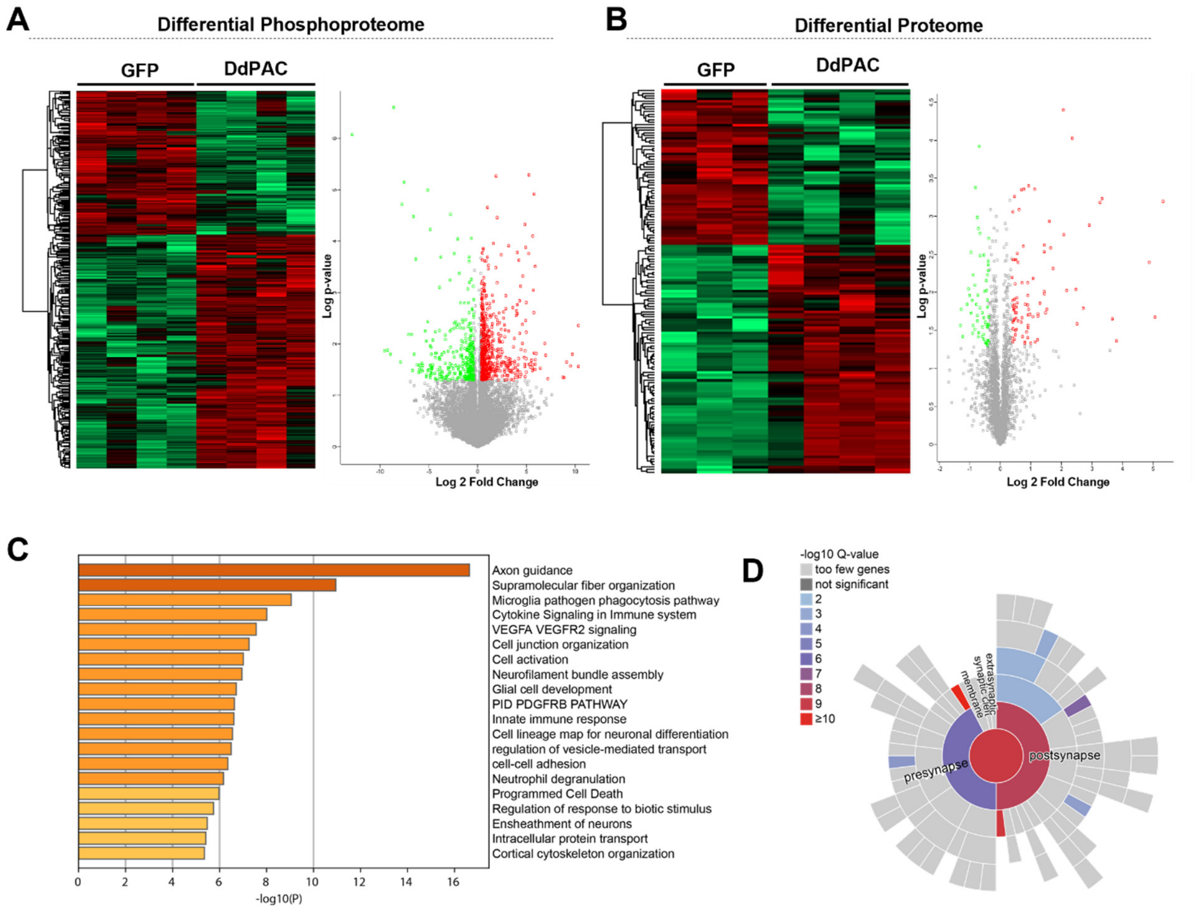

SUPPLEMENTARY FIGURE 3: Astrocytic DdPAC stimulation proteome and phosphoproteome. Effects of 10 minutes 685 nm light stimulation on freely moving WT mice expressing AAV-GFAP-DdPAC vs AAV-GFAP-GFP in the M2 cortex (n = 4 each). **A.** Differential phosphoproteome heatmap and volcano plot. **B.** Differential proteome heatmap and volcano plot **C.** Top 20 main proteome ontologies. **D.** Main sub-cellular localization of the proteome.
